# Biventricular Systolic Function and Myocardial Deformation in Liver Cirrhosis: A Systematic Review and Meta-Analysis of Speckle-Tracking Echocardiography and Cardiac Magnetic Resonance Feature Tracking Studies

**DOI:** 10.3390/jcm15135139

**Published:** 2026-07-01

**Authors:** Andrea Sonaglioni, Michele Lombardo, Giulio Francesco Gramaglia, Lorenzo Canova, Maria Grazia Rumi, Gian Luigi Nicolosi, Massimo Baravelli, Federica Cerini

**Affiliations:** 1 Division of Cardiology, IRCCS MultiMedica, 20123 Milan, Italy; michele.lombardo@multimedica.it (M.L.); massimo.baravelli@multimedica.it (M.B.); 2 Department of Emergency, Fondazione IRCSS Ca’ Granda, Ospedale Maggiore Policlinico, 20122 Milan, Italy; giulio.gramaglia@unimi.it; 3 Hepatology Unit, IRCCS MultiMedica, 20123 Milan, Italy; lorenzo.canova@multimedica.it (L.C.); mariagrazia.rumi@unimi.it (M.G.R.); federica.cerini@multimedica.it (F.C.); 4 Department of Clinical Sciences and Community Health, Dipartimento di Eccellenza 2023–2027, University of Milan, 20122 Milan, Italy; 5 Division of Cardiology, Policlinico San Giorgio, 33170 Pordenone, Italy; gianluigi.nicolosi@gmail.com

**Keywords:** liver cirrhosis, cirrhotic cardiomyopathy, myocardial deformation, myocardial strain, speckle-tracking echocardiography, cardiac magnetic resonance feature tracking, ventricular function

## Abstract

**Background:** Liver cirrhosis is frequently associated with cardiovascular abnormalities collectively referred to as cirrhotic cardiomyopathy, characterized by impaired cardiac reserve and subclinical myocardial dysfunction despite preserved conventional systolic function. Advanced myocardial deformation imaging techniques, including two-dimensional speckle-tracking echocardiography (2D-STE) and cardiac magnetic resonance feature tracking (CMR-FT), may allow earlier detection of subtle ventricular impairment. We performed a systematic review and meta-analysis to comprehensively evaluate conventional and deformation-derived indices of biventricular systolic function in cirrhotic patients. **Methods:** PubMed, Scopus, and EMBASE databases were systematically searched for observational studies evaluating myocardial systolic function in adult cirrhotic patients using 2D-STE and/or CMR-FT. Comparative meta-analyses between cirrhotic patients and controls were performed using standardized mean differences (SMDs) with 95% confidence intervals (CIs). Separate analyses were conducted for left ventricular ejection fraction (LVEF), left ventricular global longitudinal strain (LV-GLS), left ventricular global circumferential strain (LV-GCS), left ventricular global radial strain (LV-GRS), right ventricular ejection fraction (RVEF), and right ventricular global longitudinal strain (RV-GLS). Weighted pooled descriptive analyses of clinical, laboratory, echocardiographic, and CMR findings were additionally performed. **Results:** Twenty studies including 1553 cirrhotic patients and 498 controls were included, whereas 14 studies were eligible for quantitative meta-analysis. Conventional LVEF remained globally preserved and showed no significant overall difference between cirrhotic patients and controls, although CMR-based studies demonstrated mildly higher LVEF values in cirrhosis. Meta-analysis revealed no significant overall differences in LV-GLS, LV-GCS, LV-GRS, or RVEF, whereas RV-GLS was significantly reduced in cirrhotic patients. Substantial heterogeneity was observed across most deformation analyses. Meta-regression demonstrated significant associations between LV-GLS variability and age, body mass index, MELD score, diabetes prevalence, heart rate, systolic blood pressure, and software vendor. Descriptive pooled analyses demonstrated larger cardiac chamber dimensions, increased filling pressures, mildly increased pulmonary pressures, and increased extracellular volume fraction values in cirrhotic populations. **Conclusions:** Patients with liver cirrhosis exhibit preserved conventional systolic function despite evidence of subtle myocardial mechanical abnormalities, particularly involving right ventricular longitudinal mechanics and diastolic function. Advanced deformation imaging with 2D-STE and CMR-FT may improve early detection of subclinical cardiac involvement in cirrhotic cardiomyopathy.

## 1. Introduction

Liver cirrhosis is a chronic systemic disorder whose clinical consequences extend well beyond progressive hepatic injury and portal hypertension. In addition to liver-related complications, cirrhosis is frequently accompanied by cardiovascular abnormalities that may adversely affect symptoms, clinical outcomes, and perioperative management, particularly in patients undergoing liver transplantation evaluation [1,2,3]. Among these cardiac manifestations, cirrhotic cardiomyopathy (CCM) is increasingly recognized as a distinct clinical syndrome characterized by impaired cardiovascular reserve, subtle systolic and diastolic dysfunction, electrophysiological disturbances, and an attenuated myocardial response to physiological or pharmacological stress despite apparently preserved resting cardiac function [4,5,6]. Because advanced cirrhosis is typically associated with a hyperdynamic circulatory state and reduced systemic vascular resistance, conventional measures of systolic performance, especially left ventricular ejection fraction (LVEF), may remain normal even in the presence of underlying myocardial abnormalities [7,8]. Consequently, reliance on traditional echocardiographic parameters alone may lead to underrecognition of early cardiac involvement until disease progression or superimposed stressors unmask impaired myocardial reserve [9,10].

Growing interest in myocardial deformation imaging has improved the detection of subclinical cardiac dysfunction across a wide spectrum of cardiovascular and systemic disorders [11,12,13]. Two-dimensional speckle-tracking echocardiography (2D-STE) enables quantitative assessment of myocardial mechanics through evaluation of longitudinal, circumferential, and radial deformation patterns in an angle-independent manner [14]. Cardiac magnetic resonance feature tracking (CMR-FT) offers a complementary approach, combining deformation analysis with the high spatial resolution and reproducibility of cardiac magnetic resonance imaging [15]. Importantly, strain-derived indices may reveal subtle myocardial dysfunction before changes become evident using conventional volumetric parameters such as LVEF [16].

A growing number of investigations have explored myocardial deformation abnormalities in cirrhotic populations using 2D-STE and/or CMR-FT [17,18,19,20,21,22,23,24,25,26,27,28,29,30,31,32,33,34,35,36]. However, the available evidence remains heterogeneous, with conflicting results reported for both left- and right-sided ventricular function. In particular, left ventricular global longitudinal strain (LV-GLS), one of the most widely studied markers of subclinical systolic dysfunction, has shown variable behavior across studies, ranging from reduced deformation to apparently enhanced strain values that may reflect hyperdynamic hemodynamics and altered loading conditions. Data regarding left ventricular global circumferential strain (LV-GCS), left ventricular global radial strain (LV-GRS), right ventricular global longitudinal strain (RV-GLS), and right ventricular ejection fraction (RVEF) are comparatively more limited, especially in studies employing CMR-based methodologies. Interpretation of the literature is further complicated by differences in study design, patient characteristics, disease severity, imaging techniques, software platforms, and control group selection.

Although several individual studies have examined specific aspects of myocardial mechanics in cirrhosis, a comprehensive quantitative synthesis integrating conventional systolic indices and multidirectional strain parameters from both echocardiographic and CMR investigations is still unavailable. As a result, the overall effect of cirrhosis on biventricular myocardial performance and the degree of agreement between different imaging modalities remain insufficiently characterized.

Accordingly, the present systematic review and meta-analysis was designed to provide an integrated evaluation of biventricular systolic function in patients with liver cirrhosis using contemporary deformation imaging techniques. The primary objective was to compare LVEF, LV-GLS, LV-GCS, LV-GRS, RVEF, and RV-GLS between cirrhotic patients and healthy controls using evidence derived from studies employing 2D-STE and/or CMR-FT. In addition, pooled descriptive analyses of clinical characteristics, laboratory findings, echocardiographic measurements, and CMR parameters were performed to offer a broader overview of the studied populations and facilitate interpretation of myocardial mechanical abnormalities across different etiologies and stages of liver disease.

## 2. Materials and Methods

This systematic review and meta-analysis was performed according to the Preferred Reporting Items for Systematic Reviews and Meta-Analyses (PRISMA) statement [37]. The completed PRISMA checklist is available in the Supplementary Materials (Supplementary Material File S1).

Before initiation of the review process, the study protocol was prospectively registered in the International Platform of Registered Systematic Review and Meta-analysis Protocols (INPLASY) [1,2,3,4,5,6,17,18,19,20,21,22,23,24,25,26,27,28,29,30,31,32,33,34,35,36,38] (registration number INPLASY202650085; registered on 15 May 2026). The full protocol is provided in the Supplementary Materials (Supplementary Material File S2).

### 2.1. Search Strategy

A systematic search of the literature was undertaken independently by two reviewers to identify studies investigating myocardial systolic performance and deformation imaging in patients with liver cirrhosis. Three electronic databases (PubMed, Scopus, and EMBASE) were interrogated from their inception through May 2026.

The search algorithm incorporated both Medical Subject Headings (MeSH) and relevant free-text terms related to cirrhosis, myocardial mechanics, and advanced cardiac imaging. The following keywords, alone or in combination, were used: “liver cirrhosis”, “cirrhotic cardiomyopathy”, “end-stage liver disease”, “myocardial strain”, “global longitudinal strain”, “GLS”, “left ventricular strain”, “right ventricular strain”, “speckle-tracking echocardiography”, “2D-STE”, “cardiac magnetic resonance”, “CMR”, “feature tracking”, “CMR-FT”, “left ventricular ejection fraction”, and “right ventricular ejection fraction”. No limitations were imposed regarding publication year, language, or geographical origin of the studies.

Because the objective of the review was to characterize both conventional and deformation-derived measures of biventricular systolic performance, all eligible studies reporting data on LVEF, LV-GLS, LV-GCS, LV-GRS, RVEF, and/or RV-GLS were considered for data extraction. These variables were evaluated within the same search framework, and therefore no dedicated searches were conducted for individual strain indices or specific ventricular functional parameters.

To maximize study retrieval, the bibliographies of all included articles and relevant review publications were additionally examined manually. Any discrepancies arising during the screening process were resolved through discussion between the investigators, and when consensus could not be immediately reached, a third reviewer was consulted to adjudicate the final decision.

### 2.2. Eligibility Criteria

Studies were eligible for inclusion if they were observational investigations, including prospective cohorts, retrospective cohorts, or cross-sectional studies, and assessed left and/or right ventricular systolic function in adult individuals with liver cirrhosis or end-stage liver disease using 2D-STE and/or CMR-FT. To be considered for quantitative synthesis, studies had to report extractable data for at least one of the following variables: LVEF, LV-GLS, LV-GCS, LV-GRS, RVEF, or RV-GLS. Continuous data were accepted when presented as mean ± standard deviation, median with measures of dispersion, or any format allowing reliable statistical conversion.

Given the dual purpose of the review, namely quantitative comparison and descriptive characterization of cirrhotic populations, eligibility criteria were applied according to two predefined analytical frameworks. Studies including a healthy control group were incorporated into the comparative meta-analyses. Conversely, studies lacking a control population were retained for the systematic review and descriptive pooled analyses, thereby contributing to the characterization of clinical, laboratory, echocardiographic, and CMR findings in cirrhosis, but were not included in comparative effect-size calculations.

Because the review sought to evaluate myocardial mechanics across different imaging platforms, investigations employing either echocardiographic or CMR-based deformation techniques were considered suitable for inclusion. Accordingly, studies using 2D-STE, stress speckle-tracking echocardiography, or CMR-FT were eligible. When both resting and stress-derived measurements were reported, baseline values were preferentially selected for pooled analyses whenever available. Studies incorporating pharmacological stress testing were not excluded, provided that myocardial deformation was assessed using validated strain methodologies and that the reported parameters were comparable with those derived from standard deformation imaging protocols. Potential variability related to acquisition settings, imaging methodology, and loading conditions was acknowledged as a possible contributor to inter-study heterogeneity and considered during data interpretation. By contrast, studies based on deformation techniques not directly comparable with STE or CMR-FT, including strain-encoded imaging (SENC), conventional myocardial tagging without feature-tracking analysis, or other experimental approaches, were excluded.

Studies performed in children, animal models, or preclinical experimental settings were not eligible. For investigations involving mixed populations, inclusion was restricted to studies in which data from cirrhotic patients could be clearly identified and extracted separately. Reports that did not allow the isolation of cirrhosis-specific data were excluded to preserve population homogeneity and reduce the risk of classification bias. In addition, studies that predominantly focused on patients with major cardiac disorders, such as established cardiomyopathies, significant valvular heart disease, congenital heart disease, overt structural cardiac abnormalities, or severe pulmonary hypertension unrelated to liver disease, were excluded whenever these conditions could not be distinguished from the cirrhotic cohort. In contrast, common cardiovascular risk factors frequently encountered in routine clinical practice, including hypertension, diabetes mellitus, dyslipidemia, and smoking exposure, were not considered grounds for exclusion. Their prevalence was systematically recorded and subsequently reported to facilitate interpretation of potential residual confounding.

Editorials, narrative reviews, expert consensus documents, conference abstracts, case reports, guidelines, and other non-original publications were excluded. Furthermore, studies that did not provide sufficient quantitative imaging information to permit data extraction and pooled statistical analysis were not considered eligible for meta-analysis.

### 2.3. Study Selection, Data Collection, and Variable Extraction

Study identification and selection were performed independently by two reviewers. After removal of duplicate records, titles and abstracts were screened, and potentially relevant articles subsequently underwent full-text assessment according to the predefined eligibility criteria. Any discrepancies regarding study inclusion were discussed between reviewers and resolved by consensus. When required, a third investigator was consulted to reach a final decision.

Data extraction was carried out independently by two investigators using a predefined extraction template designed specifically for this review. Information collected from each study included the first author, year of publication, country of origin, study design, imaging technique, software platform used for deformation analysis, and sample size.

Whenever available, demographic and clinical characteristics were recorded, including age, sex, body mass index (BMI), body surface area (BSA), cardiovascular risk factors, smoking status, blood pressure measurements, heart rate, cirrhosis etiology, Child–Pugh classification, Model for End-Stage Liver Disease (MELD) score, prevalence of ascites, hepatic encephalopathy, hepatopulmonary syndrome, hepatorenal syndrome, gastrointestinal bleeding, and pharmacological treatment. Laboratory variables were also collected when reported and included hemoglobin, platelet count, creatinine, sodium, bilirubin, albumin, liver function tests, N-terminal pro–B-type natriuretic peptide (NT-proBNP), high-sensitivity troponin, C-reactive protein, and coagulation-related parameters.

To characterize cardiac structure and function, conventional echocardiographic measurements were extracted from both cirrhotic and control populations. These variables included interventricular septal thickness, posterior wall thickness, left ventricular end-diastolic and end-systolic diameters and volumes, relative wall thickness, left ventricular mass index, stroke volume, cardiac index, left atrial diameter, left atrial volume index, right ventricular dimensions, tricuspid annular plane systolic excursion (TAPSE), systolic pulmonary artery pressure (sPAP), the TAPSE/sPAP ratio, the transmitral E/A ratio, and the E/e′ ratio.

Particular attention was devoted to deformation imaging parameters derived from 2D-STE and/or CMR-FT. The extracted strain variables comprised LV-GLS, LV-GCS, LV-GRS, RV-GLS, right ventricular free-wall longitudinal strain (RV-FWLS), and atrial strain measurements of both the left and right atria whenever available. Measurements of left and right ventricular ejection fraction obtained by either echocardiography or CMR were additionally recorded.

When reported, cardiac magnetic resonance variables related to chamber morphology and myocardial tissue characterization were also collected. These included left and right ventricular volumes, ventricular mass, late gadolinium enhancement (LGE), native T1 and T2 mapping values, and extracellular volume fraction (ECV).

For uniformity across analyses, strain values originally expressed as negative percentages were converted to their absolute magnitude and reported as positive values throughout the descriptive tables and quantitative syntheses. This transformation was performed solely to facilitate interpretation and did not affect relative differences between groups or statistical significance. In studies where numerical values were presented only graphically, data were retrieved using dedicated digital plot extraction software. All extracted information underwent independent verification by both reviewers, and any inconsistencies were resolved through reassessment of the original publication until agreement was achieved.

### 2.4. Methodological Quality Assessment and Risk-of-Bias Evaluation

The methodological rigor of the included studies was independently assessed by two reviewers using the National Institutes of Health (NIH) Quality Assessment Tool for Observational Cohort and Cross-Sectional Studies [38]. This validated instrument evaluates multiple aspects of study design and reporting, including participant selection, population definition, exposure and outcome ascertainment, measurement reliability, management of potential confounders, statistical procedures, and completeness of reporting.

In accordance with NIH guidance, each study was examined across 14 methodological domains. Individual items were rated as “Yes”, “No”, “Cannot Determine”, “Not Reported”, or “Not Applicable”. Domains judged as “Not Applicable” were not considered in the overall assessment, whereas affirmative responses were regarded as indicators of methodological adequacy. An overall quality score was generated based on the number of criteria fulfilled by each study.

Studies meeting 9 to 14 applicable criteria were classified as having good methodological quality, those satisfying 5 to 8 criteria were considered of fair quality, and studies fulfilling fewer than 5 criteria were categorized as poor quality. In addition to the numerical score, final quality grading also incorporated reviewer appraisal of the potential impact of specific methodological weaknesses on study validity and interpretability.

Any disagreement between reviewers regarding domain ratings or overall study classification was addressed through re-examination of the original publication and resolved by consensus.

### 2.5. Statistical Analysis

To provide a descriptive overview of the populations included in the review, study-level pooled summaries were generated for both cirrhotic patients and control subjects. Continuous variables were expressed as sample size-weighted median values accompanied by weighted interquartile ranges (Q1–Q3). Because the majority of studies reported data as mean ± standard deviation, underlying distributions were assumed to be approximately normal in order to derive pooled medians and dispersion estimates from study-level information.

These descriptive summaries were intended exclusively to characterize the overall clinical, laboratory, echocardiographic, and CMR profiles of the included cohorts. They were not designed to represent patient-level inferential analyses and should therefore be interpreted as descriptive rather than comparative statistics. Accordingly, any *p* values derived from these pooled summaries are exploratory in nature and should not be considered equivalent to formal meta-analytic effect estimates. Their purpose was solely to facilitate interpretation of the study populations and provide context for the quantitative findings.

The principal quantitative analyses focused on the impact of liver cirrhosis on conventional measures of ventricular systolic function and myocardial deformation. Separate meta-analyses were conducted for LVEF, LV-GLS, LV-GCS, LV-GRS, RVEF, and RV-GLS. Differences between cirrhotic and control populations were synthesized using standardized mean differences (SMDs) and corresponding 95% confidence intervals (CIs). The SMD metric was selected because of anticipated variability in measurement scales, acquisition protocols, software platforms, and imaging modalities across studies.

Model selection was based on the magnitude of between-study heterogeneity. Random-effects models using the DerSimonian–Laird method were applied when relevant clinical or methodological variability was present, whereas fixed-effect models were adopted when heterogeneity was minimal. Heterogeneity was evaluated using Cochran’s Q test and quantified with the I^2^ statistic. Conventionally, I^2^ values of approximately 25%, 50%, and 75% were interpreted as representing low, moderate, and high heterogeneity, respectively.

The stability of pooled estimates was assessed through leave-one-out sensitivity analyses performed for each outcome. Potential publication bias and small-study effects were examined visually using funnel plots and statistically using Egger’s regression test when the number of available studies allowed meaningful assessment.

To further explore possible determinants of inter-study variability, meta-regression analyses were undertaken for outcomes analyzed using random-effects models whenever sufficient data were available. Prespecified moderators included age, sex distribution, body mass index, MELD score, Child–Pugh score, heart rate, blood pressure measurements, imaging modality, and software vendor. For each covariate, regression coefficients, standard errors, 95% confidence intervals, and two-sided *p* values were calculated. Given the relatively limited number of studies available for several models, meta-regression findings were considered exploratory and hypothesis-generating. Consequently, any statistically significant associations should be interpreted cautiously and viewed as potential contributors to heterogeneity rather than definitive causal relationships.

All analyses were performed using Comprehensive Meta-Analysis software (version 3.0; Biostat, Englewood, NJ, USA). Statistical significance was defined by a two-tailed *p* value < 0.05.

### 2.6. Use of Artificial Intelligence for Language Refinement

Artificial intelligence–based software was utilized solely to improve the linguistic quality and readability of the manuscript. Specifically, ChatGPT (OpenAI, San Francisco, CA, USA; GPT-5.5 version) was used to assist with grammar correction, stylistic refinement, sentence restructuring, and enhancement of overall language clarity.

Artificial intelligence tools did not contribute to the study conception, literature search, study selection, data extraction, quality assessment, statistical analyses, interpretation of findings, or formulation of the scientific conclusions. All methodological decisions, analytical procedures, data interpretation, and final scientific content were independently developed, critically reviewed, and approved by the authors, who take full responsibility for the integrity and accuracy of the work.

## 3. Results

### 3.1. Literature Search and Study Selection

The database search strategy yielded 919 potentially relevant records from PubMed, Scopus, and EMBASE. Following the removal of 29 duplicate entries, 890 unique citations remained and were screened on the basis of title and abstract. After the application of the predefined eligibility criteria, 836 records were excluded.

A total of 54 articles were subsequently reviewed in full text for detailed eligibility assessment. Of these, 34 studies were excluded because they did not provide sufficient STE and/or CMR-FT data for inclusion in the present analysis. Consequently, 20 studies [17,18,19,20,21,22,23,24,25,26,27,28,29,30,31,32,33,34,35,36] met all eligibility requirements and were included in the systematic review. Among these, 14 investigations incorporating an appropriate healthy control group [17,18,19,20,21,22,23,24,25,26,27,28,31,34] contributed to the quantitative meta-analysis, whereas 6 studies were retained only for descriptive synthesis because no control population was available.

**Figure 1:**
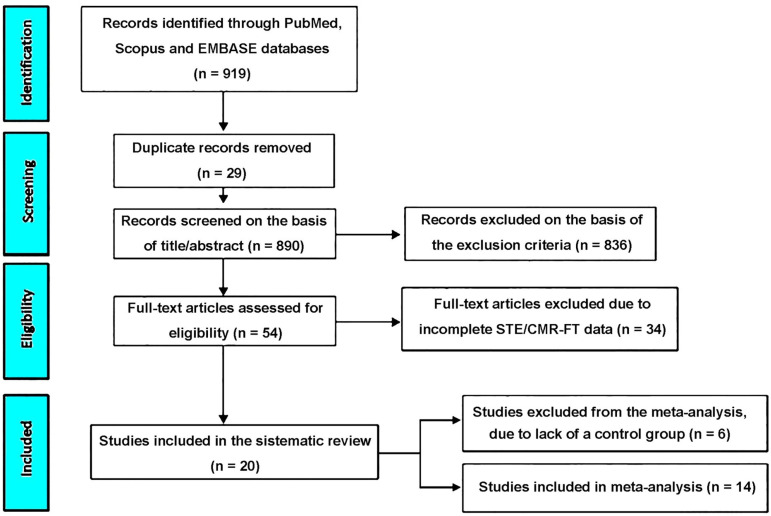
PRISMA flowchart illustrating the identification, screening, eligibility assessment, and final inclusion of studies in the systematic review and meta-analysis.

A detailed overview of the study identification, screening, eligibility assessment, and inclusion process is presented in the PRISMA flow diagram (Figure 1).

### 3.2. Overview of Included Studies and Study Populations

Twenty studies published between 2014 and 2026 satisfied the eligibility criteria and were included in the systematic review. Overall, the analyzed population comprised 1553 patients with liver cirrhosis and 498 control subjects. The studies originated from multiple geographic regions, including Europe, Asia, and North Africa, reflecting a broad representation of cirrhotic populations and clinical practice settings. A comprehensive summary of study design, patient characteristics, imaging modalities, and methodological features is provided in Table 1.

Most studies adopted a prospective single-center design and used 2D-STE as the primary imaging modality, whereas CMR-FT was employed in a smaller number of investigations. Considerable methodological heterogeneity was observed regarding study design, imaging modality, software vendor, and disease severity. Overall, the literature reflects the progressive evolution from early studies focused on left ventricular longitudinal mechanics toward more comprehensive multimodality assessments incorporating biventricular strain, atrial deformation, stress imaging, and tissue characterization.

### 3.3. Demographic and Clinical Characteristics of the Study Populations

The pooled demographic and clinical profiles of cirrhotic patients and healthy controls across the included studies are summarized in Table 2.

The included cirrhotic cohorts consisted predominantly of middle-aged men with moderate-to-advanced liver disease. The typical hemodynamic profile was consistent with hyperdynamic circulation, characterized by higher heart rate and lower blood pressure values compared with controls.

Viral and alcohol-related liver disease represented the most common etiologies. Clinical markers of portal hypertension and hepatic decompensation were frequently reported. Cardiovascular risk factors and medical therapies varied across studies, and reporting completeness differed substantially among cohorts.

### 3.4. Laboratory Characteristics of Cirrhotic Patients and Controls

The pooled laboratory characteristics of cirrhotic patients and healthy controls derived from the included studies are reported in Table 3.

Laboratory data were consistent with the typical biochemical profile of advanced liver disease, including impaired hepatic synthetic function, cytopenias, altered coagulation parameters, and increased markers of hepatocellular injury.

Compared with controls, cirrhotic cohorts generally demonstrated lower hemoglobin, platelet count, and albumin levels, together with higher bilirubin, liver enzyme, and international normalized ratio (INR) values. Available biomarker data also suggested evidence of increased cardiovascular stress and low-grade systemic inflammation.

### 3.5. Echocardiographic Structural, Functional, and Myocardial Deformation Parameters

The pooled echocardiographic profiles of cirrhotic patients and healthy controls across the included studies are summarized in Table 4.

Echocardiographic assessment of cirrhotic patients revealed mild cardiac chamber remodeling, increased left atrial volume index, higher E/e′ ratios, and mildly elevated systolic pulmonary artery pressures, consistent with early diastolic dysfunction despite preserved conventional systolic performance.

Compared with controls, LVEF and TAPSE remained largely unchanged, whereas myocardial deformation analyses suggested subtle impairment of ventricular and atrial mechanics, supporting the presence of subclinical myocardial involvement in cirrhotic cardiomyopathy.

### 3.6. Cardiac Magnetic Resonance Structural, Functional, and Tissue Characterization Findings

Table 5 presents the pooled CMR findings obtained in cirrhotic patients and control subjects.

CMR analyses provided complementary evidence of subtle myocardial functional and structural abnormalities in cirrhotic patients despite preserved global systolic performance.

Cirrhotic cohorts exhibited features consistent with a hyperdynamic circulatory state, including higher LVEF values and lower ventricular end-systolic volumes, together with increased extracellular volume fraction suggestive of diffuse myocardial interstitial remodeling. Although differences in CMR-derived strain parameters were generally modest, these findings support the presence of subclinical myocardial involvement. Nevertheless, the limited number of available CMR studies warrants cautious interpretation of these observations.

### 3.7. Comparative Analysis of Left Ventricular Ejection Fraction in Cirrhotic Patients and Healthy Controls

**Figure 2:**
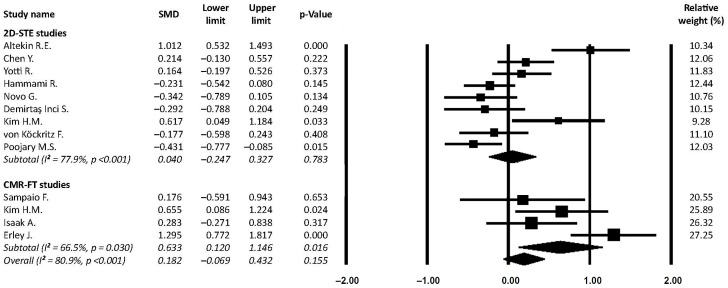
Forest plot of differences in LVEF between cirrhotic patients and healthy controls, with subgroup analysis according to imaging modality (2D-STE [17,19,20,21,22,25,26,28,31] vs. CMR-FT [18,26,27,34]).

The results of the meta-analysis evaluating differences in LVEF between patients with liver cirrhosis and healthy controls are illustrated in the forest plot (Figure 2).

Using a random-effects model, pooled analysis demonstrated no statistically significant overall difference in LVEF between cirrhotic patients and healthy subjects, with an overall SMD of 0.182 (95% CI −0.069 to 0.432; *p* = 0.155).

Subgroup analyses stratified according to imaging modality revealed important differences between echocardiographic and CMR-derived measurements. Among studies using echocardiography, pooled analysis demonstrated no significant difference in LVEF between cirrhotic patients and controls (SMD 0.040, 95% CI −0.247 to 0.327; *p* = 0.783). In contrast, studies using CMR showed significantly higher LVEF values in cirrhotic patients (SMD 0.633, 95% CI 0.120–1.146; *p* = 0.016). Between-subgroup heterogeneity was statistically significant (Q_between = 3.905, *p* = 0.048), suggesting that imaging modality substantially influenced the magnitude of observed systolic functional differences.

Substantial between-study heterogeneity was detected across the included investigations (I^2^ = 80.9%; Q = 62.939, *p* < 0.001). High levels of heterogeneity persisted in modality-specific analyses, with I^2^ values of 77.9% among echocardiographic studies and 66.5% among CMR-based studies. Despite substantial heterogeneity, the overall pooled analysis suggested the relative preservation of conventional systolic function in cirrhotic patients when assessed using LVEF.

**Figure 3:**
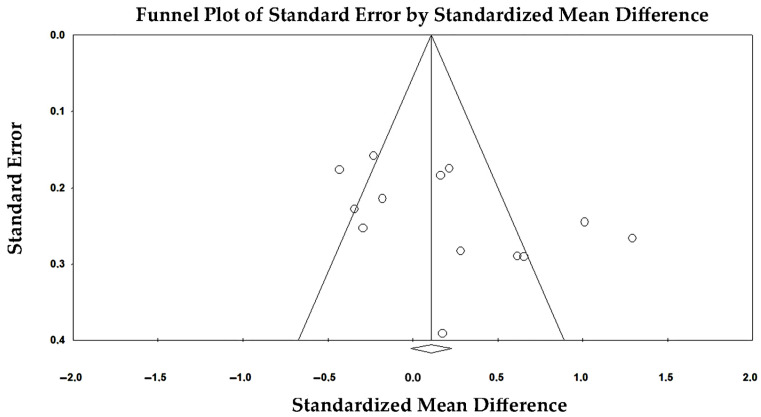
Funnel plot of studies comparing LVEF between cirrhotic patients and controls.

Potential small-study effects and publication bias were assessed through visual examination of funnel plots (Figure 3) and formal testing with Egger’s regression method.

Visual evaluation of the funnel plot suggested an overall balanced distribution of studies around the pooled effect size, although a slight predominance of positive estimates was apparent among smaller studies. Consistent with this observation, Egger’s regression analysis yielded an intercept of 4.651 (SE 2.431), without reaching statistical significance (two-tailed *p* = 0.082). These findings do not support the presence of significant publication bias, although a borderline signal of small-study effects cannot be completely excluded.

To investigate potential sources of between-study variability, random-effects meta-regression analyses were subsequently conducted. Demographic, clinical, and methodological characteristics were examined as candidate moderators of the observed differences in LVEF between cirrhotic patients and healthy controls. The corresponding results are presented in Table 6.

No significant relationship was identified between any of the examined moderator variables and the pooled effect size. However, BMI and diabetes prevalence showed borderline associations with LVEF differences (both *p* = 0.101), suggesting that metabolic factors may partially contribute to variability in conventional systolic function among cirrhotic populations. Similarly, mean age demonstrated a non-significant trend toward higher effect estimates (*p* = 0.159). No significant influence was observed for sex distribution, MELD score, heart rate, systolic blood pressure, beta-blocker therapy, alcoholic etiology, or software vendor.

Leave-one-out sensitivity analyses demonstrated the stability of the overall findings. Sequential removal of individual studies produced only modest changes in the pooled SMD estimates, without materially affecting the direction or significance of the results. Across all iterations, pooled effect sizes remained within a narrow range (approximately 0.110–0.263), and all corresponding *p* values remained above the predefined threshold for statistical significance.

### 3.8. Comparative Analysis of Left Ventricular Global Longitudinal Strain B in Cirrhotic Patients and Healthy Controls

**Figure 4:**
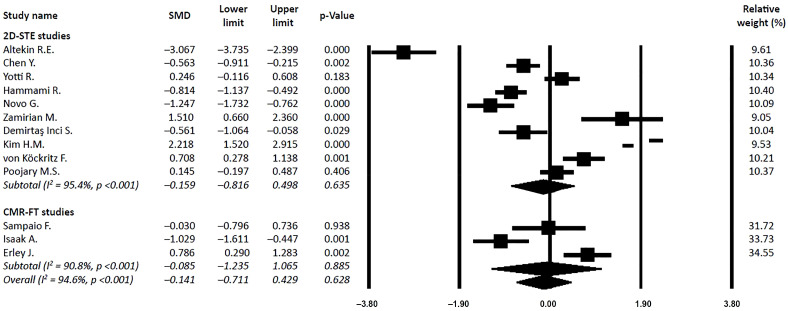
Forest plot of differences in LV-GLS between cirrhotic patients and healthy controls, with subgroup analysis stratified according to imaging modality (2D-STE [17,19,20,21,22,24,25,26,28,31] vs. CMR-FT [18,27,34]).

Figure 4 displays the forest plot summarizing the meta-analysis of LV-GLS differences between individuals with liver cirrhosis and control subjects.

Visual inspection demonstrated marked dispersion among individual study estimates, with substantial variability in both the direction and magnitude of reported LV-GLS differences. Several investigations demonstrated significantly impaired LV-GLS values in cirrhotic patients, whereas other studies reported preserved or even supranormal deformation indices compared with healthy controls.

A random-effects meta-analysis did not identify a significant difference in LV-GLS between patients with liver cirrhosis and healthy controls. The pooled effect estimate corresponded to an SMD of −0.141 (95% CI −0.711 to 0.429; *p* = 0.628). Consistent results were observed in analyses stratified according to imaging modality. Studies employing 2D-STE yielded a pooled SMD of −0.159 (95% CI −0.816 to 0.498; *p* = 0.635), whereas CMR-FT studies showed an SMD of −0.085 (95% CI −1.235 to 1.065; *p* = 0.885). No significant differences emerged between imaging subgroups (Q_between = 0.012, *p* = 0.912), indicating overall agreement between echocardiographic and CMR-based assessments.

A marked degree of between-study variability was observed. Overall heterogeneity was extremely high (I^2^ = 94.6%; Q = 220.489, *p* < 0.001), and similarly elevated values were detected within both modality-specific subgroups, including 2D-STE studies (I^2^ = 95.4%) and CMR-FT studies (I^2^ = 90.8%). These findings highlight substantial variability in the reported effects across individual investigations.

Although the pooled analysis did not demonstrate a significant overall difference, the direction and magnitude of LV-GLS findings varied considerably among studies. Individual reports described patterns ranging from reduced longitudinal deformation suggestive of subclinical systolic impairment to apparently augmented strain values, potentially reflecting the hyperdynamic circulatory state that characterizes advanced liver disease.

**Figure 5:**
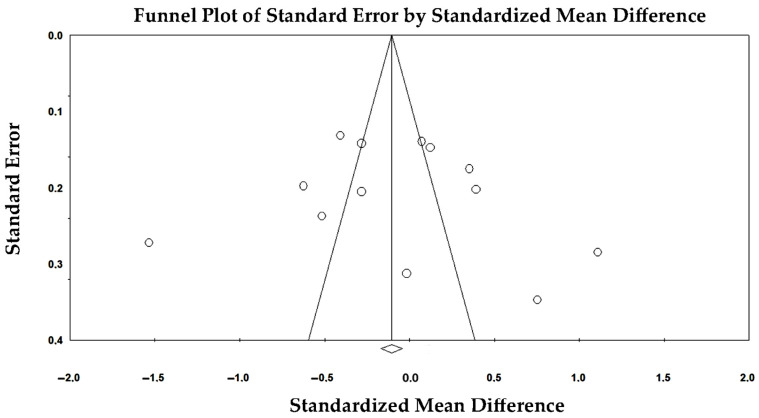
A funnel plot illustrating the distribution of studies included in the meta-analysis of LV-GLS differences between patients with liver cirrhosis and healthy controls, used to examine potential publication bias and small-study effects.

Publication bias and potential small-study effects were subsequently explored through visual assessment of the funnel plot (Figure 5) and Egger’s regression analysis.

Visual examination of the funnel plot revealed a generally balanced distribution of studies around the pooled effect size, with no obvious indication of substantial asymmetry. Consistent with this observation, Egger’s regression analysis yielded an intercept of 1.492 (SE 4.258), and the corresponding two-sided *p* value was not statistically significant (*p* = 0.733). These findings do not support the presence of meaningful publication bias or relevant small-study effects.

To further explore potential determinants of the observed inter-study variability, random-effects meta-regression analyses were conducted. Demographic characteristics, clinical variables, and methodological factors were evaluated as possible moderators of LV-GLS differences between cirrhotic patients and healthy controls. The results of these analyses are summarized in Table 7.

Several covariates demonstrated statistically significant associations with pooled LV-GLS effect size. Greater age, higher BMI, and increased heart rate were associated with lower LV-GLS effect estimates, whereas higher MELD score, diabetes prevalence, systolic blood pressure, and use of non-GE software were associated with higher effect sizes. No significant associations were observed for sex distribution, alcoholic etiology, or beta-blocker therapy.

The robustness of the pooled estimates was supported by leave-one-out sensitivity analyses. Sequential exclusion of individual studies resulted in only minor changes in effect size, with recalculated SMDs ranging from approximately −0.331 to 0.082. In all iterations, *p* values remained non-significant, indicating that no individual study exerted a substantial influence on the overall findings.

### 3.9. Comparative Analysis of Left Ventricular Global Circumferential Strain in Cirrhotic Patients and Healthy Controls

**Figure 6:**
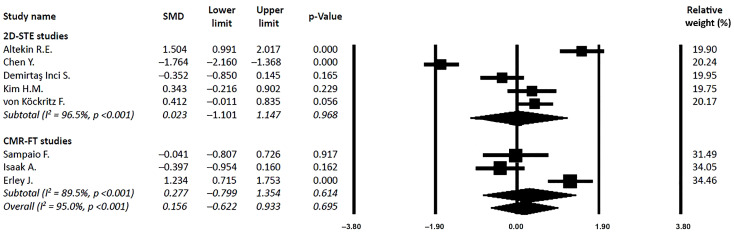
Forest plot of differences in LV-GCS between cirrhotic patients and healthy controls, with subgroup analysis stratified according to imaging modality (2D-STE [17,19,25,26,28] vs. CMR-FT [18,27,34]).

The results of the meta-analysis evaluating differences in LV-GCS between patients with liver cirrhosis and healthy controls are presented in the forest plot (Figure 6).

Visual inspection demonstrated pronounced variability in individual study estimates, with several investigations reporting opposite directions of effect. Some studies demonstrated significantly impaired LV-GCS values in cirrhotic patients, whereas others reported preserved or increased circumferential deformation compared with healthy controls.

The random-effects meta-analysis did not demonstrate a significant difference in LV-GCS between cirrhotic patients and healthy controls. The pooled effect estimate corresponded to an SMD of 0.156 (95% CI −0.622 to 0.933; *p* = 0.695). Consistent results were obtained in analyses stratified according to imaging modality. In studies using 2D-STE, the pooled SMD was 0.023 (95% CI −1.101 to 1.147; *p* = 0.968), whereas CMR-FT studies yielded an SMD of 0.277 (95% CI −0.799 to 1.354; *p* = 0.614). Comparison between modality-specific subgroups revealed no significant heterogeneity (Q_between = 0.103, *p* = 0.749), supporting overall agreement between echocardiographic and CMR-derived assessments of circumferential deformation.

A substantial degree of inter-study variability was evident across the included investigations. Overall heterogeneity was very high (I^2^ = 95.0%; Q = 139.946, *p* < 0.001), and similarly elevated values were observed within both the 2D-STE subgroup (I^2^ = 96.5%) and the CMR-FT subgroup (I^2^ = 89.5%). The broad distribution of individual study estimates highlights the marked variability in reported LV-GCS findings among cirrhotic cohorts.

**Figure 7:**
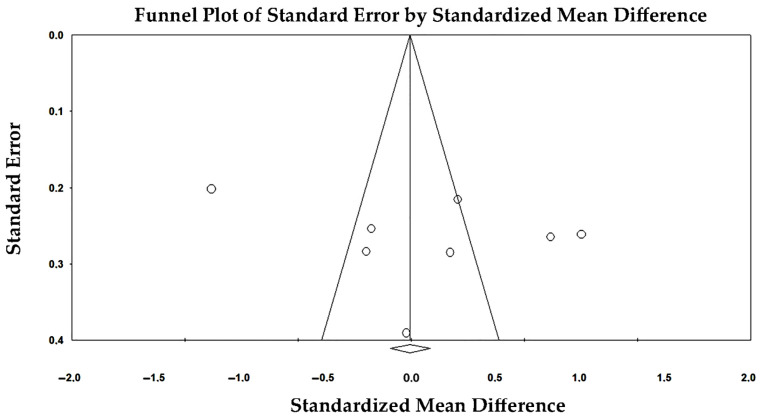
Funnel plot of studies comparing LV-GCS between cirrhotic and control populations.

The possibility of publication bias was assessed using both visual inspection of the funnel plot (Figure 7) and Egger’s regression analysis for funnel plot asymmetry.

Visual examination of the funnel plot revealed a generally balanced distribution of studies around the pooled effect size, without obvious evidence of substantial asymmetry. Consistent with this observation, Egger’s regression analysis produced an intercept of 9.009 (SE 9.032), with a non-significant two-sided *p* value (*p* = 0.357). Overall, these findings do not suggest the presence of significant publication bias or meaningful small-study effects.

To investigate possible contributors to the observed between-study heterogeneity, random-effects meta-regression analyses were subsequently performed. Demographic and methodological variables were examined as potential moderators of LV-GCS differences between cirrhotic patients and healthy controls. The corresponding results are reported in Table 8.

Meta-regression analyses did not identify any significant moderator of the pooled LV-GCS effect estimate. In particular, no meaningful association was observed for age, sex distribution, MELD score, heart rate, or software vendor, suggesting that these variables did not explain the substantial heterogeneity observed across studies.

The robustness of the pooled findings was further supported by leave-one-out sensitivity analyses. Sequential omission of individual studies resulted in only limited variation in the pooled SMD estimates, without materially altering the overall interpretation of the results. Recalculated effect sizes ranged from approximately −0.086 to 0.396, and all corresponding *p* values remained above the predefined threshold for statistical significance. These findings indicate that the overall results were not driven by any single study.

### 3.10. Comparative Analysis of Left Ventricular Global Radial Strain in Cirrhotic Patients and Healthy Controls

**Figure 8:**
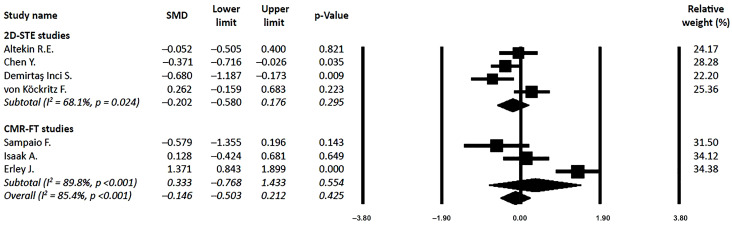
Forest plot summarizing the meta-analysis of LV-GRS differences between patients with liver cirrhosis and healthy controls. Subgroup analyses were performed according to imaging modality, including 2D-STE [17,19,25,28] and CMR-FT [18,27,34].

The pooled analysis investigating differences in LV-GRS between cirrhotic patients and healthy controls is reported in the forest plot (Figure 8).

Inspection of individual study estimates revealed substantial variability in both magnitude and direction of effect sizes. While some investigations demonstrated lower LV-GRS values in cirrhotic patients, other studies reported preserved or even higher radial strain values compared with controls.

The random-effects meta-analysis did not demonstrate a significant difference in LV-GRS between cirrhotic patients and healthy controls. The pooled effect estimate corresponded to an SMD of −0.146 (95% CI −0.503 to 0.212; *p* = 0.425). Comparable results were observed in subgroup analyses stratified according to imaging modality. Studies using 2D-STE yielded a pooled SMD of −0.202 (95% CI −0.580 to 0.176; *p* = 0.295), whereas CMR-FT studies showed an SMD of 0.333 (95% CI −0.768 to 1.433; *p* = 0.554). No significant difference was detected between modality-specific subgroups (Q_between = 0.811, *p* = 0.368), indicating overall consistency between echocardiographic and CMR-derived measurements.

Substantial between-study heterogeneity was observed across the included investigations. The overall heterogeneity estimate was high (I^2^ = 85.4%; Q = 41.136, *p* < 0.001) and remained considerable within both imaging subgroups, with I^2^ values of 68.1% for 2D-STE studies and 89.8% for CMR-FT studies. The marked variability in individual study estimates underscores the heterogeneous nature of reported radial myocardial deformation findings in patients with liver cirrhosis.

**Figure 9:**
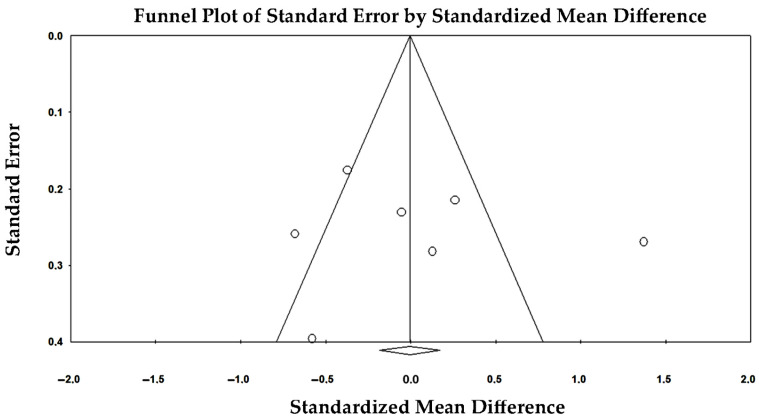
Funnel plot of studies comparing LV-GRS between cirrhotic and control populations.

Publication bias and potential small-study effects were subsequently explored through visual evaluation of the funnel plot (Figure 9) and formal testing with Egger’s regression method.

Visual examination of the funnel plot revealed a generally balanced distribution of studies around the pooled effect size, with no obvious indication of substantial asymmetry. In agreement with this observation, Egger’s regression analysis yielded a non-significant intercept (1.427, SE 4.888; two-sided *p* = 0.782), providing no evidence of meaningful publication bias or relevant small-study effects.

To further investigate potential sources of between-study heterogeneity, random-effects meta-regression analyses were performed. Demographic and methodological characteristics were evaluated as possible moderators of LV-GRS differences between cirrhotic patients and healthy controls. The results of these analyses are presented in Table 9.

Meta-regression analyses did not identify any statistically significant association between LV-GRS effect size and the evaluated study-level covariates. In particular, age, BMI, MELD score, and software vendor did not significantly contribute to between-study variability.

Leave-one-out sensitivity analyses confirmed the robustness of the overall results. Th removal of individual studies one at a time produced only minor fluctuations in the pooled SMD estimates, with effect sizes ranging from approximately −0.190 to 0.137. Across all iterations, *p* values remained non-significant, indicating that no single study exerted a substantial influence on the pooled effect estimate.

### 3.11. Comparative Analysis of Right Ventricular Ejection Fraction in Cirrhotic Patients and Healthy Controls

**Figure 10:**
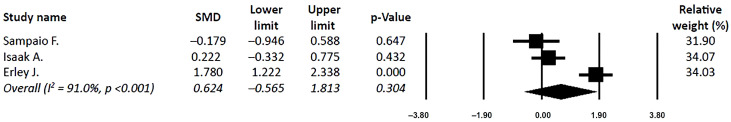
Forest plot comparing CMR-derived RVEF between patients with liver cirrhosis and healthy control subjects across the included studies [18,27,34].

The pooled analysis evaluating differences in RVEF between cirrhotic patients and healthy controls is presented in the forest plot (Figure 10).

Individual studies reported heterogeneous findings regarding RVEF. Two investigations found no significant differences between cirrhotic patients and healthy controls, whereas Erley et al. [34] reported significantly higher RVEF values in the cirrhotic cohort.

The random-effects meta-analysis did not demonstrate a significant overall difference in RVEF between groups. The pooled effect estimate corresponded to an SMD of 0.624 (95% CI −0.565 to 1.813; *p* = 0.304). Although the direction of the pooled estimate favored higher RVEF values in cirrhotic patients, the wide confidence intervals encompassed the null value, precluding definitive conclusions.

A high degree of between-study heterogeneity was observed (I^2^ = 91.0%; Q = 22.205, *p* < 0.001), indicating substantial variability across the included investigations despite the use of the same imaging modality. Inspection of individual study results suggests that this heterogeneity was largely attributable to the findings reported by Erley et al. [34], which differed markedly from the remaining studies by demonstrating substantially higher RVEF values among patients with liver cirrhosis.

**Figure 11:**
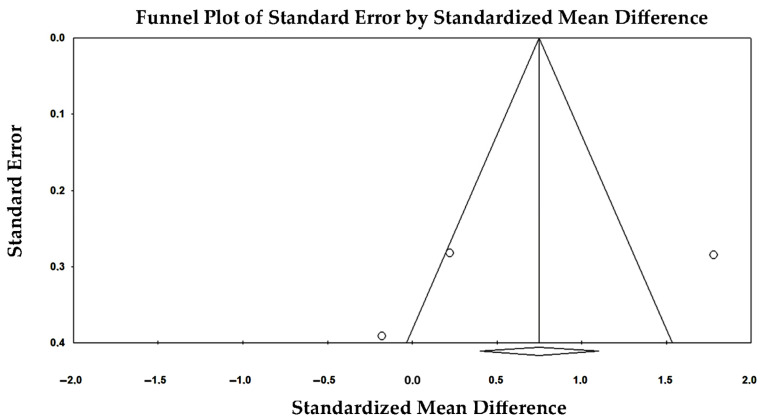
Funnel plot illustrating the distribution of studies included in the meta-analysis of RVEF in cirrhotic patients versus healthy controls.

Assessment of publication bias was performed by examining funnel plot symmetry (Figure 11) and applying Egger’s regression method.

Visual examination of the funnel plot revealed no clear evidence of asymmetry. Nevertheless, the interpretation of this finding should be approached cautiously because only a limited number of studies were available for analysis. In agreement with the graphical assessment, Egger’s regression test did not identify significant small-study effects or publication bias (intercept = −10.504, SE = 16.109; two-sided *p* = 0.632).

Because only three studies contributed data to this analysis and all employed CMR feature-tracking methodology, no meta-regression analyses were undertaken.

Leave-one-out sensitivity analyses were performed to evaluate the robustness of the pooled estimate. Sequential omission of individual studies resulted in some variation in effect size magnitude. The largest change was observed after exclusion of the study by Erley et al. [34], which reduced the pooled SMD from 0.624 to 0.085. Conversely, the removal of either the study by Sampaio et al. [18] or that by Isaak et al. [27] led to numerically greater pooled estimates. Despite these fluctuations, none of the recalculated models reached statistical significance, supporting the overall stability of the primary findings.

Across all iterations, pooled SMD values ranged approximately between 0.085 and 0.819, with corresponding *p*-values consistently remaining above the significance threshold. These findings suggest that the overall results were influenced by individual study estimates, particularly the investigation by Erley et al. [34], although no single study was sufficient to generate a statistically significant pooled effect.

### 3.12. Comparative Analysis of Right Ventricular Global Longitudinal Strain in Cirrhotic Patients and Healthy Controls

**Figure 12:**
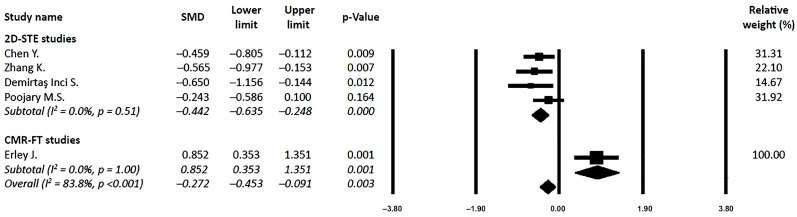
Forest plot comparing RV-GLS between cirrhotic patients and healthy controls, including subgroup analyses stratified according to imaging technique (2D-STE [19,23,25,31] and CMR-FT [34]).

The forest plot summarizing the pooled comparison of RV-GLS between cirrhotic individuals and healthy control subjects is presented in Figure 12.

Most studies employing 2D-STE demonstrated lower RV-GLS values in cirrhotic patients compared with controls, whereas the single CMR-FT study reported significantly higher RV-GLS values in the cirrhotic population.

Pooled quantitative synthesis performed using a fixed-effects model identified a significant difference in RV-GLS between patients with liver cirrhosis and healthy control subjects. The overall effect estimate favored lower RV-GLS values in cirrhotic patients (SMD −0.272, 95% CI −0.453 to −0.091; *p* = 0.003).

When analyses were stratified according to imaging modality, studies using 2D speckle-tracking echocardiography consistently demonstrated impaired right ventricular longitudinal mechanics in cirrhosis, yielding a pooled SMD of −0.442 (95% CI −0.635 to −0.248; *p* < 0.001). Notably, no significant heterogeneity was detected among these investigations (I^2^ = 0.0%; *p* = 0.515). Conversely, the only study employing CMR feature tracking reported significantly greater RV-GLS values in cirrhotic individuals compared with controls (SMD 0.852, 95% CI 0.353–1.351; *p* = 0.001).

Considerable heterogeneity emerged across the overall analysis (I^2^ = 83.8%; Q = 24.707, *p* < 0.001). This variability appeared largely driven by the discordant findings observed between echocardiographic and CMR-based assessments. Consistent with this interpretation, heterogeneity attributable to subgroup differences was highly significant (Q_between = 22.421, *p* < 0.001), supporting a substantial effect of imaging modality on the estimated RV-GLS differences.

Despite the high heterogeneity observed in the overall model, results from the 2D-STE subgroup were remarkably homogeneous, with nearly all studies indicating worse right ventricular longitudinal deformation in cirrhotic patients than in healthy controls.

**Figure 13:**
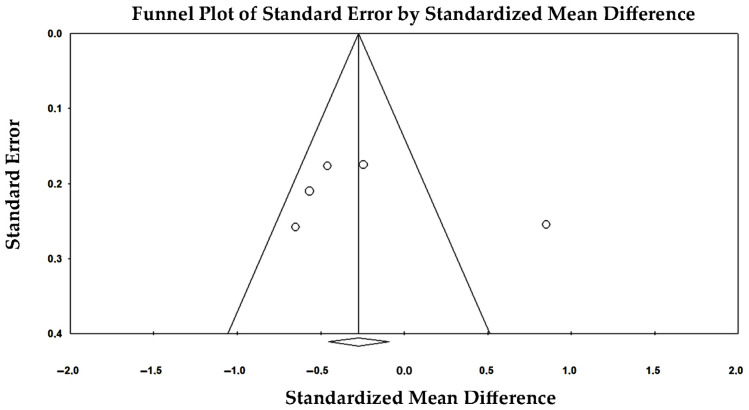
Graphical assessment of publication bias for studies investigating RV-GLS differences between individuals with liver cirrhosis and healthy controls.

Possible publication bias was subsequently explored through visual evaluation of the funnel plot (Figure 13) together with Egger’s regression analysis.

Inspection of the funnel plot did not suggest marked asymmetry. However, the limited number of studies available for analysis restricts the reliability of visual interpretation. Consistent with this observation, Egger’s regression test showed a non-significant intercept of 4.900 (SE 7.262; two-sided *p* = 0.548), providing no statistical indication of publication bias or meaningful small-study effects.

Meta-regression was not undertaken because of the small evidence base and the availability of only one study using CMR feature-tracking methodology, which precluded meaningful exploration of potential moderators.

The robustness of the pooled findings was further evaluated through leave-one-out sensitivity analyses. Sequential omission of individual studies resulted in only modest changes in the magnitude of the pooled effect estimate, while statistical significance was preserved in nearly all recalculated models. Across the different iterations, pooled SMD values remained within a relatively narrow range, varying from approximately −0.442 to −0.202, thereby supporting the overall stability of the observed association.

### 3.13. Methodological Quality of the Included Studies

The methodological rigor of the included investigations [17,18,19,20,21,22,23,24,25,26,27,28,29,30,31,32,33,34,35,36] was evaluated using the NIH Quality Assessment Tool for Observational Cohort and Cross-Sectional Studies [38]. A comprehensive summary of the quality assessment results is provided in the Supplementary Materials (Supplementary Material File S3).

The overall methodological standard of the included studies was judged to be satisfactory. Most investigations provided clear descriptions of the study population, eligibility criteria, imaging acquisition protocols, and outcome definitions. In addition, the application of established 2D-STE and/or CMR-FT methodologies was generally consistent across the analyzed literature. Methodological quality was further enhanced in several studies through the use of matched control cohorts, multivariable statistical adjustment, serial evaluations over time, and blinded image interpretation.

Based on the prespecified NIH quality classification system, the majority of studies achieved a rating of good methodological quality, while a smaller number were categorized as fair. Only one investigation was considered to have poor methodological quality, largely owing to a limited sample size, insufficient reporting of participant recruitment procedures, absence of blinded assessments, and inadequate control of potential confounding factors.

Common methodological weaknesses identified across the literature included the lack of formal power analyses, incomplete documentation of blinding procedures, the absence of repeated exposure measurements, and restricted follow-up duration in studies with cross-sectional designs. Despite these limitations, no substantial concerns were identified regarding the validity of outcome measurements or the overall coherence of the reported data.

Taken together, the quality assessment supports the methodological credibility of the available evidence and indicates that the included studies provide a reasonably robust foundation for the present systematic review and meta-analysis.

## 4. Discussion

### 4.1. Principal Findings

The present systematic review and meta-analysis provides a comprehensive multimodality evaluation of conventional systolic function and myocardial deformation abnormalities in patients with liver cirrhosis assessed by 2D-STE and CMR-FT. Overall, our findings support the idea that cirrhotic cardiomyopathy is characterized by complex and heterogeneous myocardial mechanical alterations that may remain clinically silent despite preserved conventional systolic indices.

An important finding of the present analysis is that conventional left ventricular systolic function assessed by LVEF appeared globally preserved across most cirrhotic populations, with pooled analyses demonstrating no significant overall difference compared with healthy controls despite the hyperdynamic circulatory profile commonly observed in advanced liver disease. However, CMR-based studies tended to report slightly higher LVEF values in cirrhotic patients, likely reflecting the combined effects of reduced afterload, increased preload, and enhanced sympathetic activation characterizing cirrhosis-related hemodynamic dysfunction.

Myocardial deformation analysis revealed markedly heterogeneous findings across studies and imaging modalities. Although pooled LV-GLS and LV-GCS meta-analyses did not demonstrate statistically significant overall differences between cirrhotic patients and controls, individual investigations showed wide variability ranging from impaired myocardial deformation to apparently preserved or even supranormal strain values. This heterogeneity was evident both in the direction and magnitude of the reported effects, with some studies demonstrating reduced strain values in cirrhotic patients, whereas others reported preserved or even enhanced myocardial deformation. These findings likely reflect the multifactorial pathophysiology of cirrhotic cardiomyopathy, in which myocardial fibrosis, chronic inflammation, autonomic dysfunction, altered loading conditions, and hyperdynamic circulation may simultaneously influence deformation indices in different directions. Part of this heterogeneity may also be attributable to methodological differences between 2D-STE and CMR-FT. Compared with echocardiography, CMR-FT generally provides superior spatial resolution but lower temporal resolution and relies on different feature-tracking approaches, potentially resulting in systematic differences in strain magnitude and reproducibility. Consequently, direct quantitative comparisons between deformation values obtained with the two modalities should be interpreted cautiously.

Beyond myocardial deformation, pooled echocardiographic and CMR analyses consistently demonstrated evidence of subtle structural and functional remodeling despite preserved conventional systolic performance. Cirrhotic patients exhibited larger left ventricular dimensions and volumes, increased left atrial volume index, higher filling pressures estimated by E/e′, and mildly increased sPAP values. In addition, right ventricular longitudinal mechanics appeared mildly impaired, supporting the idea that cirrhosis may involve both left- and right-sided myocardial function.

CMR tissue characterization findings further suggested the presence of diffuse myocardial interstitial remodeling in cirrhotic populations. Increased extracellular volume fraction and numerically higher native T1 and late gadolinium enhancement values support the hypothesis that subclinical myocardial fibrosis and low-grade inflammatory remodeling may contribute to the pathophysiological substrate of cirrhotic cardiomyopathy even before overt systolic dysfunction becomes clinically evident.

Another relevant observation was the substantial between-study heterogeneity detected across most pooled analyses. Meta-regression suggested that demographic, hemodynamic, metabolic, and methodological factors—including age, BMI, MELD score, diabetes prevalence, heart rate, systolic blood pressure, and software vendor—may partially explain the dispersion of strain findings across studies. The inclusion of investigations employing different deformation imaging modalities may have further contributed to this heterogeneity, supporting the need for modality-specific interpretation of the pooled results. Nevertheless, sensitivity analyses consistently demonstrated stable pooled estimates without evidence of major publication bias, supporting the overall robustness of the present findings despite the methodological heterogeneity and limitations of the available literature.

### 4.2. Pathophysiological Pathways Linking Liver Cirrhosis to Biventricular Cardiac Dysfunction

The cardiovascular effects of liver cirrhosis should be interpreted as the result of a progressive interaction between portal hypertension, systemic vasodilation, neurohormonal activation, inflammatory signaling, and myocardial structural remodeling [39]. Chronic liver injury and fibrotic distortion of hepatic architecture increase intrahepatic vascular resistance, promoting portal hypertension and portosystemic shunting [40,41]. This process facilitates the systemic spillover of vasodilatory mediators, including nitric oxide and other endothelial-derived factors, leading to splanchnic and peripheral arterial vasodilation, reduced systemic vascular resistance, relative central hypovolemia, and compensatory activation of the sympathetic nervous system and renin–angiotensin–aldosterone system [42,43]. The resulting hyperdynamic circulation, characterized by increased heart rate and cardiac output with reduced arterial pressure, may preserve or even enhance conventional systolic indices at rest, thereby masking early myocardial dysfunction [44,45]. Importantly, this hyperdynamic state profoundly alters ventricular loading conditions, increasing preload while simultaneously reducing systemic afterload. As myocardial deformation indices are highly load-dependent parameters, strain behavior in cirrhosis may dynamically fluctuate according to the balance between circulatory compensation and intrinsic myocardial impairment. Consequently, apparently preserved or even supranormal strain values do not necessarily exclude early myocardial dysfunction in advanced liver disease. Taken together, these observations suggest that the hyperdynamic circulation of cirrhosis could, at least in part, represent a dynamic compensatory response rather than a fixed hemodynamic phenotype, although this hypothesis requires confirmation in prospective mechanistic studies. Over time, progressive impairment of cardiac reserve may compromise this compensatory response, thereby contributing to circulatory dysfunction, refractory ascites, renal hypoperfusion, hepatorenal syndrome, and ultimately increased mortality [46,47,48].

This hemodynamic adaptation, however, occurs at the cost of chronic cardiovascular stress. Persistent adrenergic stimulation and renin–angiotensin–aldosterone system (RAAS) activation promote sodium and water retention, plasma volume expansion, chamber dilation, and progressive myocardial remodeling [49]. Chronic autonomic dysfunction, characterized by sustained sympathetic overactivation and impaired β-adrenergic responsiveness, may further contribute to progressive myocardial energetic inefficiency and reduced cardiac reserve. Over time, this maladaptive neurohormonal state may promote myocardial oxygen demand–supply mismatch, relative myocardial ischemia, and worsening ventricular mechanical performance. In this setting, LVEF may remain normal because of reduced afterload and increased preload, whereas myocardial deformation indices may reveal impaired contractile efficiency, particularly under stress conditions. This explains why cirrhotic cardiomyopathy is often clinically silent at rest but may become evident during exercise, dobutamine stimulation, hemorrhage, sepsis, transjugular intrahepatic portosystemic shunt (TIPS) implantation, or liver transplantation [50,51,52]. Consistently, stress-based studies showed that cirrhotic patients may have blunted increases in cardiac output, ejection fraction, and strain reserve despite apparently preserved resting function [18,24,53].

At the myocardial level, several mechanisms may directly impair contractility. Experimental and clinical data support abnormalities in β-adrenergic receptor signaling, altered membrane fluidity and ion channel function, impaired calcium handling, and increased exposure to cardiodepressant mediators [54,55,56]. Systemic inflammation may further contribute through bacterial translocation, release of pathogen-associated molecular patterns, tumor necrosis factor-α, interleukin pathways, nitric oxide overproduction, and oxidative stress [57,58]. These mechanisms may induce myocardial inflammation, reduce contractile responsiveness, and contribute to the dissociation between preserved conventional systolic function and impaired myocardial deformation. Yotti et al. [20] specifically linked left ventricular systolic indices to sympathetic activity and inflammatory markers, supporting the idea that myocardial performance in cirrhosis is dynamically modulated by neuroinflammatory activation rather than solely by intrinsic contractility. Importantly, systemic inflammation, autonomic dysregulation, and altered loading conditions likely interact synergistically to modulate myocardial deformation behavior. This complex pathophysiological interplay may partly explain the marked heterogeneity of strain findings observed across cirrhotic populations and disease stages.

CMR studies provide additional support for a structural myocardial substrate underlying cirrhotic cardiomyopathy. Increased native T1 and T2 relaxation times, higher extracellular volume fraction, and more frequent non-ischemic late gadolinium enhancement suggest diffuse interstitial expansion, edema, low-grade inflammation, and fibrosis [26,27,34,59,60]. Importantly, the severity of myocardial abnormalities appears to parallel liver disease severity, with higher ECV, T1/T2 values, and LGE burden being in more advanced Child–Pugh classes. These findings indicate that cirrhosis-related cardiac dysfunction is not only functional or load-dependent, but may also involve progressive myocardial tissue remodeling. Such structural alterations may progressively transform an initially functional and potentially reversible cardiomyopathy into a more advanced phenotype characterized by diffuse interstitial fibrosis, impaired ventricular compliance, reduced myocardial reserve, and increasing susceptibility to hemodynamic stressors.

The heterogeneous behavior of LV strain observed across studies likely reflects the balance between these competing mechanisms. Longitudinal strain, largely reflecting subendocardial fiber function, may be particularly vulnerable to fibrosis, inflammation, altered microvascular perfusion, and increased wall stress [61]. Conversely, circumferential and radial mechanics may be preserved or enhanced as compensatory mechanisms, especially in hyperdynamic states [62,63]. This may explain why some studies reported impaired LV-GLS, whereas others observed preserved or supranormal deformation values in patients with advanced cirrhosis. Therefore, strain values in this population should not be interpreted as pure markers of myocardial contractility but rather as load-sensitive indices integrating intrinsic myocardial function, chamber geometry, preload, afterload, and systemic circulatory status. Accordingly, myocardial strain in cirrhosis should be interpreted within the broader hemodynamic and pathophysiological context rather than as an isolated surrogate of intrinsic systolic function. This concept is particularly relevant in advanced cirrhosis, where profound preload/afterload alterations may partially mask underlying myocardial contractile impairment.

Right heart involvement represents an equally important component of cirrhosis-related cardiac dysfunction [64]. Increased blood volume, fluid retention, pulmonary vascular changes, hepatopulmonary syndrome, portopulmonary vascular abnormalities, and elevated pulmonary pressures may increase right ventricular (RV) loading conditions and impair RV–pulmonary arterial coupling. Although conventional RV systolic parameters often remain preserved, RV longitudinal strain and right atrial strain may detect early impairment of right-sided mechanics. Importantly, among all deformation parameters analyzed in the present meta-analysis, RV-GLS represented the most consistently impaired systolic index in cirrhotic populations, suggesting that the right ventricle may be particularly vulnerable to the hemodynamic and pulmonary vascular consequences of advanced liver disease. This finding is pathophysiologically plausible, as the thin-walled RV is highly sensitive to chronic preload increase, pulmonary vascular abnormalities, and subtle elevations in pulmonary arterial pressure, even before overt RV systolic dysfunction becomes detectable using conventional indices such as TAPSE or RVEF. Despite these observations, firm conclusions remain difficult because of the restricted number of studies, limited cohort sizes, and the likelihood that methodological differences between imaging modalities may influence RV strain estimates. From a pathophysiological perspective, progressive impairment of RV longitudinal mechanics may reflect early RV–pulmonary arterial uncoupling, a phenomenon increasingly recognized as an important determinant of exercise intolerance, reduced cardiac reserve, and adverse clinical outcomes in several cardiopulmonary diseases [65]. Zhang et al. [23] showed that RV and RA remodeling may be present in end-stage cirrhosis despite preserved conventional RV systolic indices, with correlations between MELD score, liver enzymes, bilirubin, and right heart deformation parameters. Current evidence is insufficient to draw definitive conclusions regarding the role of RV-GLS in cirrhotic cardiomyopathy. Larger prospective cohorts, together with greater methodological standardization of image acquisition and strain analysis, will be essential to determine the reliability, clinical applicability, and long-term prognostic impact of right ventricular deformation abnormalities in this setting.

**Figure 14:**
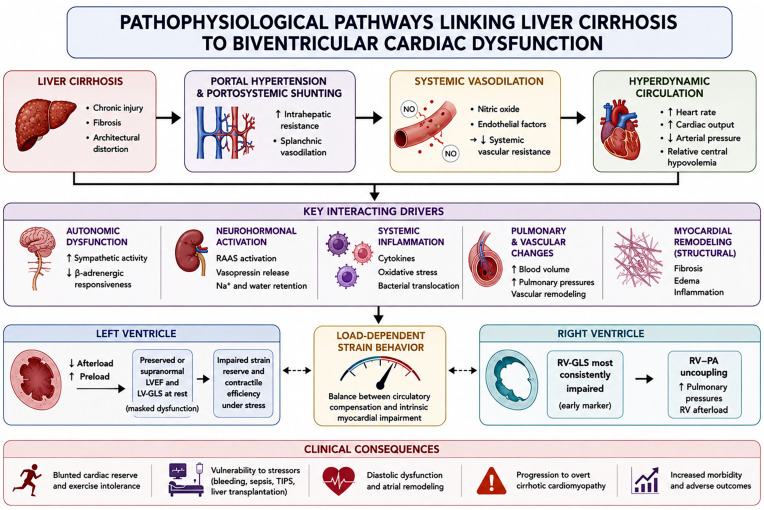
Schematic representation of the complex pathophysiological mechanisms underlying cirrhosis-related cardiac dysfunction. Portal hypertension and portosystemic shunting promote systemic vasodilation and hyperdynamic circulation, resulting in increased preload and reduced afterload. These hemodynamic alterations interact with autonomic dysfunction, neurohormonal activation, systemic inflammation, pulmonary vascular abnormalities, and progressive myocardial remodeling. Such mechanisms may contribute to preserved or supranormal conventional systolic indices despite impaired myocardial reserve and subclinical ventricular dysfunction. The left ventricle may exhibit apparently preserved LVEF and LV-GLS at rest because of altered loading conditions, whereas the right ventricle appears particularly vulnerable to chronic preload increase and pulmonary vascular involvement, with RV-GLS emerging as an early marker of RV–pulmonary arterial uncoupling. Progressive myocardial fibrosis, impaired ventricular compliance, and reduced cardiac reserve may ultimately lead to exercise intolerance, stress-induced cardiac dysfunction, overt cirrhotic cardiomyopathy, and adverse clinical outcomes.

Overall, the pathophysiological substrate of biventricular dysfunction in cirrhosis is best understood as a continuum. In early or compensated stages, reduced afterload and hyperdynamic circulation may maintain or increase LVEF, RVEF, and some strain parameters. With disease progression, persistent neurohormonal activation, systemic inflammation, myocardial fibrosis, chamber remodeling, pulmonary vascular involvement, and impaired cardiac reserve progressively reduce the capacity of both ventricles to respond to hemodynamic stress. This framework explains the apparently paradoxical findings of preserved or supranormal resting systolic indices coexisting with impaired deformation, abnormal tissue characterization, diastolic dysfunction, atrial remodeling, exercise intolerance, reduced hemodynamic reserve, and adverse outcomes in advanced liver disease (Figure 14).

### 4.3. Potential Clinical and Imaging Implications

The present findings have several potential clinical implications for the cardiovascular assessment of patients with liver cirrhosis. Conventional systolic indices, particularly resting LVEF, often remain preserved despite evidence of subclinical myocardial abnormalities. In contrast, myocardial deformation imaging by 2D-STE or CMR-FT may provide a more sensitive assessment of early cardiac involvement [66]. However, strain values obtained by these modalities should not be interpreted interchangeably because of important technical differences, including temporal resolution, feature-tracking methodology, post-processing algorithms, and vendor-specific variability [67,68]. Consequently, serial evaluations should preferably be performed using the same imaging modality and software platform.

The detection of impaired myocardial deformation may be particularly relevant in patients exposed to hemodynamic stressors such as TIPS implantation, major surgery, gastrointestinal bleeding, sepsis, or liver transplantation. Under these conditions, latent myocardial dysfunction may become clinically evident despite apparently preserved resting systolic function. Accordingly, deformation imaging may complement conventional echocardiographic assessment in selected clinical settings, although its incremental prognostic value and optimal role in pre-procedural risk stratification remain to be established.

Our findings further support the idea that cirrhotic cardiomyopathy extends beyond isolated LV systolic dysfunction. The combined involvement of ventricular and atrial mechanics highlights the potential value of a comprehensive multiparametric assessment integrating ventricular strain, atrial function, diastolic indices, chamber remodeling, and, when available, CMR tissue characterization [69,70].

The substantial heterogeneity observed across studies also indicates that myocardial deformation parameters should always be interpreted within their hemodynamic context. In particular, apparently preserved or even supranormal strain values may reflect the hyperdynamic circulatory state characteristic of advanced cirrhosis rather than intrinsically normal myocardial function [71].

**Figure 15:**
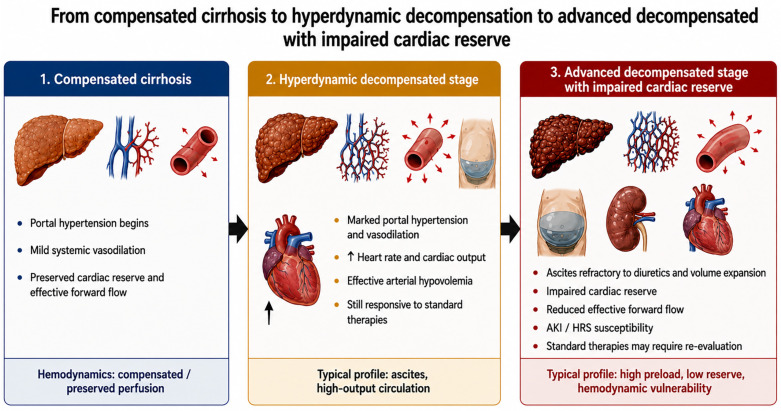
Conceptual framework illustrating the proposed progression from compensated cirrhosis to advanced decompensated stages characterized by hyperdynamic circulatory dysfunction, impaired cardiac reserve, and increased susceptibility to circulatory and cardiorenal complications.

From a broader pathophysiological perspective, the available evidence may support the existence of a progressive transition from compensated cirrhosis to advanced decompensated stages characterized by impaired cardiac reserve, reduced circulatory adaptability, and increasing cardiovascular vulnerability. This conceptual framework should therefore be regarded as hypothesis-generating and may provide a useful model for integrating the complex interplay among hyperdynamic circulation, chronic neurohormonal activation, myocardial remodeling, and the heightened susceptibility to circulatory and renal complications observed in advanced liver disease (Figure 15).

Recent evidence has also demonstrated early impairment of LV mechanics in patients with MASLD despite preserved LVEF, suggesting that myocardial deformation imaging may have value across the broader spectrum of chronic liver disease and not only in established cirrhosis [72]. In addition, the potentially reversible nature of at least part of cirrhosis-related myocardial dysfunction raises the possibility that strain imaging could be useful for longitudinal assessment. However, dedicated prospective studies are required to determine its value for follow-up, risk re-stratification, and monitoring of therapeutic response.

Taken together, these findings reinforce the importance of multidisciplinary collaboration between hepatologists, cardiologists, cardiac imagers, anesthesiologists, and transplant teams when evaluating patients with advanced liver disease.

Importantly, the present meta-analysis was not designed to establish diagnostic thresholds, prognostic cut-off values, or specific clinical algorithms for the use of myocardial deformation parameters before TIPS implantation, liver transplantation, or other interventions. Therefore, the clinical implications discussed above should be considered exploratory and hypothesis-generating.

### 4.4. Determinants of Inter-Study Variability, Study Strengths, and Methodological Limitations

A major finding of the present meta-analysis was the substantial heterogeneity observed across most pooled analyses, particularly for myocardial deformation parameters derived from both 2D-STE and CMR-FT studies. This variability likely reflects the multifactorial nature of cirrhotic cardiomyopathy together with important differences in cirrhosis etiology, disease severity, loading conditions, cardiovascular comorbidities, pharmacological treatments, and imaging methodologies among the included investigations. Advanced liver disease is characterized by hyperdynamic circulation, reduced systemic vascular resistance, sodium and fluid retention, neurohormonal and RAAS activation, systemic inflammation, endothelial dysfunction, and myocardial interstitial remodeling, all of which may variably influence myocardial deformation indices independently of intrinsic myocardial contractility. Although the MELD score was reported in many studies and remains central for liver disease severity assessment and liver transplantation prioritization, it likely provides only a partial representation of the overall pathophysiological burden of decompensated cirrhosis. Indeed, the severity of portal hypertension, systemic circulatory dysfunction, and cardiodynamic impairment may not necessarily parallel the degree of biochemical liver dysfunction. Consequently, clinically relevant hemodynamic and cardiovascular risk may already be present despite relatively modest MELD values and therefore may not be fully captured by MELD alone.

Importantly, the included studies were highly heterogeneous with respect to patient selection, cirrhosis etiology, liver disease severity, prevalence of cardiovascular comorbidities, imaging modality, software vendor, acquisition protocols, and study design. Such variability may have substantially influenced both conventional and deformation-derived parameters and should be considered when interpreting the pooled estimates. Therefore, the summary effect sizes reported in the present meta-analysis should be interpreted as reflecting overall trends across heterogeneous cirrhotic populations rather than uniform pathophysiological effects consistently applicable across all stages and etiologies of liver disease.

The present study has several strengths. To the best of our knowledge, this is the first systematic review and meta-analysis to comprehensively integrate conventional systolic indices, multidirectional myocardial deformation parameters, and CMR tissue characterization findings in patients with liver cirrhosis using both echocardiographic and CMR-based techniques. The multimodality approach allowed a comprehensive characterization of biventricular myocardial mechanics across different clinical settings and disease severities, while sensitivity analyses, publication bias assessment, and meta-regression models further strengthened the robustness of the pooled findings.

Nevertheless, several limitations should be acknowledged. Most included studies were single-center observational investigations with relatively small sample sizes, and several analyses relied on study-level rather than individual patient-level data. In addition, the number of available CMR-FT studies and the reporting of advanced tissue characterization parameters remained limited. Importantly, STE-derived strain measurements are intrinsically influenced by image quality, frame rate optimization, endocardial border delineation, motion artifacts, and inter-vendor variability, all factors that may substantially affect tracking reproducibility and temporal resolution [73,74,75]. Furthermore, extrinsic mechanical factors such as thoracic geometry, body habitus, and anterior chest wall conformation may alter myocardial motion tracking independently of intrinsic myocardial dysfunction [76,77]. Myocardial strain parameters are also highly load-dependent indices [78], and therefore the hyperdynamic circulatory state characteristic of advanced cirrhosis may significantly influence deformation measurements independently of true myocardial contractile impairment. Several limitations specific to CMR-FT should also be considered, including lower temporal resolution compared with echocardiography, potential inaccuracies related to through-plane motion, inter-vendor variability in post-processing algorithms, and lower reproducibility of radial strain measurements. Finally, the limited availability, higher costs, and lower accessibility of CMR currently restrict the widespread clinical implementation of CMR-FT in routine practice [79].

## 5. Conclusions

Patients with liver cirrhosis exhibit complex cardiovascular alterations characterized by preserved conventional systolic function despite evidence of subtle abnormalities in myocardial mechanics, chamber remodeling, and diastolic function.

Advanced deformation imaging techniques, including 2D-STE and CMR-FT, may improve the detection of subclinical myocardial involvement in cirrhotic cardiomyopathy, particularly when conventional volumetric indices remain normal. Among deformation parameters, alterations in right ventricular longitudinal mechanics emerged as one of the most consistent findings across the available studies, whereas left ventricular deformation results were more heterogeneous and appeared to be influenced by loading conditions, disease severity, imaging modality, and methodological variability.

Further large prospective multimodality studies are warranted to better define the diagnostic and prognostic significance of myocardial deformation imaging and to clarify the clinical relevance and longitudinal evolution of myocardial mechanical abnormalities in patients with liver cirrhosis.

## Figures and Tables

**Table 1:** Summary of the main methodological and clinical characteristics of studies included in the systematic review and meta-analysis [17,18,19,20,21,22,23,24,25,26,27,28,29,30,31,32,33,34,35,36].

Study Name, Publication Year and Country	Study Design	Method	Software	Sample Size (% Male)	MELD Score
Altekin R.E. (2014), Turkey [17]	Prospective, single-center	2D-STE	GE	38 (63.2%)	11.8
Sampaio F. (2015), Portugal [18]	Prospective, single-center	Dobutamine stress CMR-FT	Circle Cardiovascular Imaging Inc.	36 (83.3%)	9
Chen Y. (2016), China [19]	Prospective, single-center	2D-STE	GE	103 (74.8%)	NS
Yotti R. (2017), Spain [20]	Prospective, single-center	2D-STE	GE	59 (76.0%)	13
Hammami R. (2017), Tunisia [21]	Cross-sectional, single-center	2D-STE	GE	80 (52.5%)	14.2
Novo G. (2018), Italy [22]	Prospective, single-center	2D-STE	Siemens	39 (41.0%)	7
Zhang K. (2019), Germany [23]	Retrospective, single-center	2D-STE	GE	67 (49.0%)	13.5
Zamirian M. (2019), Iran [24]	Prospective, single-center	2D-STE + dobutamine stress echo	GE	20 (50.0%)	NS
Demirtaş Inci S. (2019), Turkey [25]	Prospective, multi-center	2D-STE	Siemens	40 (70.0%)	NS
Kim H.M. (2020), South Korea [26]	Prospective, single-center	2D-STE and CMR-FT	TomTec Imaging Systems	33 (75.8%)	18.8
Isaak A. (2020), Germany [27]	Prospective, multi-center	CMR-FT	TomTec Imaging Systems	42 (55.0%)	12.4
von Köckritz F. (2021), Germany [28]	Retrospective, single-center	2D-STE	GE	80 (58.8%)	17
Soulaidopoulos S. (2021), Greece [29]	Prospective, single-center	2D-STE	GE	130 (72.3%)	14.6
Jansen C. (2022), Germany [30]	Retrospective, single-center	2D-STE	TomTec Imaging Systems	206 (61.1%)	11.3
Poojary M.S. (2022), India [31]	Prospective, single-center	2D-STE	GE	70 (81.4%)	15.7
Luo Y. (2023), China [32]	Prospective, single-center	2D-STE	NS	104 (82.7%)	17.7
Skouloudi M. (2023), Greece [33]	Prospective, single-center	2D-STE	Philips	135 (65.2%)	14
Erley J. (2025), Germany [34]	Prospective, single-center	CMR-FT	Circle Cardiovascular Imaging Inc.	50 (54.0%)	14
Yang X. (2026), China [35]	Cross-sectional, single-center	2D-STE	GE	122 (72.0%)	11.7
Radu T. (2026), Romania [36]	Retrospective, single-center	2D-STE	GE	99 (66.6%)	14.6

The table reports the study design, imaging modality, and software vendor used for myocardial deformation analysis; the study sample size with the percentage of male participants; and the mean MELD score when available.

**Table 2:** Descriptive pooled clinical profile of patients with liver cirrhosis and healthy controls based on weighted study-level data.

Clinical Parameters	Studies Reporting Each Variable (n° Cirrhotics vs. Controls)	Cirrhotics	Controls	Exploratory *p* Value
Age	20 vs. 14 (1553 vs. 498)	55.2 (53–57)	52.2 (48.6–55)	<0.001
Males (%)	20 vs. 14 (1553 vs. 498)	66.6 (61.1–74.8)	55 (46.7–66.7)	<0.001
BSA (m^2^)	5 vs. 3 (361 vs. 69)	1.9 (1.9–2)	1.9 (1.8–1.9)	<0.001
BMI (Kg/m^2^)	13 vs. 7 (1215 vs. 233)	25.5 (24.5–26.4)	24.9 (24–26.6)	0.012
Hypertension (%)	5 vs. 2 (360 vs. 87)	14 (12.6–25.2)	14.5 (14.5–41)	<0.001
Smoking (%)	4 vs. 2 (296 vs. 87)	11.7 (11.7–32.7)	16.7 (2.6–16.7)	<0.001
Diabetes (%)	5 vs. 2 (360 vs. 87)	20 (19.3–20.4)	10.4 (10.4–20.5)	<0.001
Dyslipidemia (%)	2 vs. 1 (89 vs. 39)	8 (5.1–8)	12.8 (12.8–12.8)	<0.001
HR (bpm)	15 vs. 9 (1152 vs. 377)	75 (72–76.9)	74 (67–74.4)	<0.001
QTc (msec)	4 vs. 3 (256 vs. 139)	437 (420.6–437)	349 (349–410)	<0.001
SBP (mmHg)	11 vs. 6 (785 vs. 240)	118.3 (118–120)	121 (119.1–136)	<0.001
DBP (mmHg)	10 vs. 6 (655 vs. 240)	68 (67–76)	76 (73.5–76.5)	<0.001
Viral (%)	10 vs. 0 (921 vs. 0)	31.5 (24.4–52.6)	/	/
Alcoholic (%)	10 vs. 0 (879 vs. 0)	37.6 (27.4–58.3)	/	/
Autoimmune (%)	1 vs. 0 (33 vs. 0)	6.1 (6.1–6.1)	/	/
MASLD/NASH (%)	1 vs. 0 (135 vs. 0)	7.4 (7.4–7.4)	/	/
PSC/PBC (%)	1 vs. 0 (135 vs. 0)	17 (17–17)	/	/
Cryptogenic (%)	3 vs. 0 (248 vs. 0)	8.9 (8.9–26.1)	/	/
Other causes (%)	7 vs. 0 (719 vs. 0)	24.7 (21.2–24.7)	/	/
Child–Pugh score	9 vs. 0 (833 vs. 0)	7.9 (7.5–8.4)	/	/
MELD score	17 vs. 0 (1390 vs. 0)	14 (11.7–14.6)	/	/
Ascites (%)	5 vs. 0 (590 vs. 0)	67.7 (58–70.4)	/	/
HCC (%)	2 vs. 0 (265 vs. 0)	10.4 (10.4–11.5)	/	/
Hepatopulmonary syndrome (%)	1 vs. 0 (130 vs. 0)	34.6 (34.6–34.6)	/	/
History of variceal bleeding (%)	4 vs. 0 (332 vs. 0)	27.6 (27.6–46.2)	/	/
GI bleeding (%)	3 vs. 0 (338 vs. 0)	23 (9.6–24.2)	/	/
History of encephalopathy (%)	7 vs. 0 (855 vs. 0)	22.8 (19.3–33.8)	/	/
Hepatorenal Syndrome (%)	1 vs. 0 (206 vs. 0)	23.8 (23.8–23.8)	/	/
Spontaneous bacterial peritonitis (%)	3 vs. 0 (369 vs. 0)	15.6 (8.4–19.2)	/	/
BB (%)	5 vs. 0 (357 vs. 0)	67.4 (56–67.4)	/	/
Diuretics (%)	4 vs. 0 (277 vs. 0)	54 (53–64.4)	/	/
Spironolactone (%)	1 vs. 0 (59 vs. 0)	66 (66–66)	/	/
ACEi/ARBs (%)	2 vs. 0 (83 vs. 0)	8 (8–9.1)	/	/
Statins (%)	1 vs. 0 (50 vs. 0)	2 (2–2)	/	/

Continuous and categorical variables are presented as sample size-weighted median values together with weighted interquartile ranges (Q1–Q3). Variables related to cirrhosis etiology, disease severity, complications, and medical therapy, which were available exclusively in cirrhotic cohorts, are summarized descriptively and were not subjected to formal comparisons with control populations. The reported exploratory *p* values were calculated from pooled study-level descriptive summaries and are provided solely to facilitate the interpretation of cohort characteristics. These values should not be regarded as patient-level inferential statistics, nor should they be interpreted as quantitative effect estimates derived from meta-analytic models.

**Table 3:** Summary of laboratory parameters expressed as weighted medians and weighted interquartile ranges (Q1–Q3) for cirrhotic and control populations.

Laboratory Parameters	Studies Reporting Each Variable (n° Cirrhotics vs. Controls)	Cirrhotics	Controls	Exploratory *p* Value
Hb (g/dL)	7 vs. 2 (629 vs. 70)	10.73 (10.14–11.27)	13.43 (13.30–13.98)	<0.001
PLTs (×10^9^/L)	4 vs. 1 (385 vs. 8)	115.98 (96.77–129.50)	225.00 (225.00–225.00)	<0.001
Creatinine (mg/dL)	12 vs. 7 (829 vs. 209)	0.94 (0.80–1.16)	0.80 (0.78–0.87)	<0.001
Sodium (mmol/L)	4 vs. 2 (371 vs. 70)	138.00 (137.61–138.38)	138.19 (137.70–140.34)	<0.001
Bilirubin (mg/dL)	11 vs. 5 (811 vs. 173)	1.90 (1.25–3.47)	0.59 (0.48–0.81)	<0.001
Albumin (g/dL)	11 vs. 4 (788 vs. 117)	3.20 (3.10–3.80)	4.22 (4.00–4.43)	<0.001
AST (U/L)	9 vs. 5 (703 vs. 175)	62.22 (44.40–80.57)	27.14 (23.77–27.99)	<0.001
ALT (U/L)	10 vs. 5 (742 vs. 175)	40.90 (26.00–56.94)	25.01 (23.11–25.09)	<0.001
AP (U/L)	3 vs. 3 (167 vs. 76)	230.82 (221.78–578.42)	66.09 (65.84–66.26)	<0.001
GGT (U/L)	5 vs. 3 (433 vs. 103)	124.42 (100.94–148.38)	22.20 (21.60–23.68)	<0.001
Fasting glucose (mg/dL)	1 vs. 1 (40 vs. 26)	97.00 (97.00–97.00)	90.00 (90.00–90.00)	N/A
Total cholesterol (mg/dL)	1 vs. 1 (40 vs. 26)	114.00 (114.00–114.00)	155.00 (155.00–155.00)	N/A
Triglycerides (mg/dL)	1 vs. 1 (40 vs. 26)	79.00 (79.00–79.00)	130.00 (130.00–130.00)	N/A
HS-troponin (pg/mL)	2 vs. 0 (185 vs. 0)	6.86 (4.76–8.00)	NR	N/A
NT-proBNP (pg/mL)	5 vs. 1 (447 vs. 8)	113.15 (105.03–211.41)	32.00 (32.00–32.00)	<0.001
CRP (mg/dL)	3 vs. 1 (284 vs. 8)	0.15 (0.10–0.24)	0.16 (0.16–0.16)	0.007
PT (INR)	9 vs. 4 (668 vs. 137)	1.30 (1.15–1.50)	0.98 (0.97–1.00)	<0.001

Continuous variables were pooled using sample size-weighted descriptive statistics derived from available study-level data. Data not available in the corresponding comparison group are indicated as NR, whereas N/A denotes variables for which formal descriptive comparison was not feasible because of unavailable control data, limited reporting, or a lack of an appropriate comparison group. Reported *p* values are exploratory and were calculated from pooled study-level descriptive summaries. These values are intended solely to facilitate descriptive interpretation and do not represent patient-level inferential comparisons or meta-analytic measures of effect.

**Table 4:** Descriptive summary of echocardiographic parameters in cirrhotic and non-cirrhotic populations based on weighted pooled data.

Echocardiographic Parameters	Studies Reporting Each Variable (n° Cirrhotics vs. Controls)	Cirrhotics	Controls	Exploratory *p* Value
IVS (mm)	9 vs. 7 (710 vs. 240)	9.73 (9.27–10.72)	9.41 (8.74–9.96)	0.079
PW (mm)	6 vs. 4 (514 vs. 143)	8.86 (8.10–10.00)	9.81 (8.00–9.93)	0.326
LVEDD (mm)	11 vs. 6 (955 vs. 225)	47.00 (44.28–47.94)	45.00 (44.91–45.00)	0.003
LVESD (mm)	5 vs. 3 (435 vs. 112)	30.26 (29.18–31.70)	28.40 (28.40–30.00)	0.223
RWT	6 vs. 4 (514 vs. 143)	0.41 (0.38–0.44)	0.41 (0.35–0.44)	0.701
LVMi (g/m^2^)	8 vs. 7 (561 vs. 319)	104.55 (79.48–110.04)	96.32 (76.54–98.47)	0.073
LVEDV (mL)	9 vs. 7 (757 vs. 301)	89.89 (88.18–94.74)	74.42 (69.49–85.05)	0.001
LVESV (mL)	8 vs. 7 (718 vs. 301)	31.25 (30.15–37.98)	27.53 (23.39–32.93)	0.053
LVEF (%)	15 vs. 9 (1338 vs. 401)	63.38 (59.71–66.01)	62.82 (60.99–64.10)	0.840
SV (mL)	4 vs. 2 (318 vs. 96)	60.38 (55.80–64.62)	45.85 (41.00–56.42)	0.80
CI (L/min/m^2^)	4 vs. 2 (318 vs. 96)	2.37 (2.30–2.58)	2.30 (2.30–2.32)	0.175
E/A	13 vs. 8 (1093 vs. 362)	1.10 (0.99–1.20)	1.20 (1.03–1.25)	0.037
E/e’	14 vs. 9 (1132 vs. 401)	8.56 (7.46–10.14)	6.58 (5.49–7.99)	<0.001
LA diameter (mm)	6 vs. 3 (470 vs. 105)	38.36 (33.07–39.74)	34.00 (34.00–34.00)	0.084
LAVi (mL/m^2^)	10 vs. 5 (850 vs. 188)	29.72 (26.90–37.48)	20.54 (16.23–23.06)	<0.001
RV basal diameter (mm)	5 vs. 2 (516 vs. 62)	31.21 (26.60–33.52)	28.25 (27.00–32.12)	0.955
TAPSE (mm)	6 vs. 3 (618 vs. 123)	23.95 (22.11–24.79)	23.00 (23.00–23.29)	0.874
sPAP (mmHg)	9 vs. 6 (861 vs. 235)	25.74 (20.42–29.70)	22.19 (17.54–25.31)	0.033
TAPSE/sPAP (mm/mmHg)	1 vs. 0 (99 vs. 0)	0.89 (0.89–0.89)	NR	N/A
LV-GLS (%)	16 vs. 10 (1358 vs. 411)	19.76 (18.46–22.07)	21.06 (19.51–21.84)	0.115
LV-GCS (%)	5 vs. 5 (294 vs. 161)	18.36 (16.60–23.18)	19.88 (17.75–22.24)	0.922
LV-GRS (%)	4 vs. 4 (261 vs. 141)	39.9 (35.9–45.4)	44.9 (44.9–52.7)	0.02
LAScd (%)	3 vs. 1 (314 vs. 30)	16.72 (15.40–19.78)	21.10 (21.10–21.10)	N/A
LASct (%)	3 vs. 1 (314 vs. 30)	17.03 (15.80–18.46)	18.80 (18.80–18.80)	N/A
LASr (%)	3 vs. 1 (314 vs. 30)	32.90 (32.90–37.21)	40.00 (40.00–40.00)	N/A
RV-GLS (%)	5 vs. 4 (410 vs. 172)	21.13 (19.76–22.40)	22.40 (21.64–23.77)	0.044
RV-FWLS (%)	2 vs. 1 (202 vs. 36)	25.24 (23.40–27.97)	26.50 (26.50–26.50)	N/A
RAVi (mL/m^2^)	1 vs. 1 (67 vs. 36)	23.00 (23.00–23.00)	17.20 (17.20–17.20)	N/A
RAScd (%)	1 vs. 1 (67 vs. 36)	20.00 (20.00–20.00)	27.00 (27.00–27.00)	NA
RASct (%)	1 vs. 1 (67 vs. 36)	19.00 (19.00–19.00)	19.00 (19.00–19.00)	N/A
RASr (%)	1 vs. 1 (67 vs. 36)	39.00 (39.00–39.00)	46.00 (46.00–46.00)	N/A

Continuous variables are expressed as sample size-weighted medians and corresponding weighted interquartile ranges (Q1–Q3). Parameters unavailable in the corresponding reference group are reported as NR, whereas N/A indicates variables for which descriptive between-group evaluation could not be reliably performed owing to insufficient comparative data, incomplete reporting, or the absence of an appropriate control population. The *p* values presented are exploratory in nature and originate from weighted descriptive estimates across studies. Accordingly, they should not be regarded as formal statistical comparisons between patient groups or as quantitative meta-analytic results.

**Table 5:** Aggregated cardiac magnetic resonance characteristics of cirrhotic and control cohorts, reported as weighted pooled estimates from the included studies.

CMR Parameters	Studies Reporting Each Variable (n° Cirrhotics vs. Controls)	Cirrhotics	Controls	Exploratory *p* Value
LVEDV (mL)	4 vs. 4 (161 vs. 71)	142.61 (135.71–152.65)	144.47 (135.00–147.22)	0.466
LVESV (mL)	4 vs. 4 (161 vs. 71)	50.10 (44.64–53.16)	53.50 (50.49–57.58)	0.022
LVEF (%)	4 vs. 4 (161 vs. 71)	65.77 (63.00–66.92)	60.42 (59.60–62.23)	0.001
SV (mL)	2 vs. 2 (83 vs. 45)	101.63 (100.00–105.62)	85.30 (85.00–86.65)	0.095
CI (L/min/m^2^)	2 vs. 2 (75 vs. 38)	3.70 (3.70–3.96)	2.92 (2.90–3.11)	0.095
LAVi (mL/m^2^)	3 vs. 3 (128 vs. 51)	47.36 (44.90–50.18)	31.38 (31.30–33.29)	<0.001
LVMi (g/m^2^)	3 vs. 3 (111 vs. 46)	48.64 (45.00–57.25)	50.94 (43.00–64.67)	0.840
RVEDV (mL)	3 vs. 3 (128 vs. 51)	153.33 (152.00–156.77)	150.06 (150.00–154.21)	0.248
RVESV (mL)	3 vs. 3 (128 vs. 51)	58.40 (54.20–65.71)	66.50 (63.95–69.75)	0.023
RVEF (%)	3 vs. 3 (128 vs. 51)	56.61 (56.00–60.20)	54.66 (54.00–55.78)	0.102
LV-GLS (%)	3 vs. 3 (128 vs. 51)	18.74 (18.50–19.26)	17.12 (17.00–20.02)	0.661
LV-GCS (%)	3 vs. 3 (128 vs. 51)	21.30 (20.90–22.69)	17.58 (17.40–22.26)	0.550
LV-GRS (%)	3 vs. 3 (128 vs. 51)	34.67 (32.80–39.27)	31.48 (31.40–33.46)	0.028
RV-GLS (%)	1 vs. 1 (50 vs. 25)	25.80 (25.80–25.80)	21.20 (21.20–21.20)	N/A
RV-GCS (%)	1 vs. 1 (50 vs. 25)	11.90 (11.90–11.90)	10.50 (10.50–10.50)	N/A
RV-GRS (%)	1 vs. 1 (50 vs. 25)	20.90 (20.90–20.90)	18.70 (18.70–18.70)	N/A
LGE (%)	2 vs. 2 (75 vs. 38)	3.32 (3.00–4.66)	0.00 (0.00–0.76)	0.095
T1 relaxation time (msec)	3 vs. 3 (125 vs. 63)	1144.67 (1008.00–1229.69)	1103.80 (958.00–1197.61)	0.816
T2 relaxation time (msec)	2 vs. 2 (92 vs. 43)	48.30 (48.30–53.14)	47.20 (47.20–49.54)	0.543
ECV (%)	3 vs. 3 (125 vs. 63)	28.74 (27.00–32.21)	25.78 (25.40–26.37)	0.044

Continuous data are summarized using weighted medians and weighted interquartile ranges (Q1–Q3), with the study sample size used as the weighting factor. N/A denotes variables for which a meaningful descriptive comparison could not be undertaken because of limited data availability, incomplete reporting, or lack of a suitable control cohort. Exploratory *p* values were generated from pooled descriptive study-level data and are provided exclusively for contextual interpretation. They do not constitute patient-level hypothesis-testing analyses or meta-analytic effect size estimates.

**Table 6:** Random-effects meta-regression exploring demographic, clinical, and methodological factors potentially contributing to differences in LVEF between patients with liver cirrhosis and healthy controls. Regression coefficients indicate the estimated variation in effect size associated with each moderator. For all covariates, 95% confidence intervals (95% CIs) and corresponding *p* values are provided. GE was used as the reference category for analyses involving software vendor.

Covariate	Coefficient	Standard Error	95% Lower	95% Upper	*p*-Value
**Intercept**	−21.277	17.628	−55.828	13.274	0.227
**Software: Non-GE**	0.297	0.713	−1.102	1.695	0.678
**Age**	0.473	0.336	−0.186	1.132	0.159
**Males**	0.038	0.033	−0.026	0.102	0.241
**BMI**	2.159	1.317	−0.423	4.741	0.101
**Alcoholic etiology**	0.011	0.033	−0.055	0.076	0.750
**MELD score**	−0.141	0.148	−0.430	0.148	0.339
**Diabetes**	−0.642	0.391	−1.409	0.125	0.101
**HR**	0.072	0.081	−0.086	0.231	0.371
**SBP**	−0.428	0.335	−1.084	0.229	0.201
**BB**	−0.003	0.027	−0.056	0.051	0.923

**Table 7:** Random-effects meta-regression evaluating demographic, clinical, and methodological factors potentially associated with variability in LV-GLS differences between cirrhotic and control populations. Regression coefficients indicate the estimated effect-size change attributable to each moderator. Ninety-five percent confidence intervals (95% CIs) and corresponding *p* values are provided. GE was used as the reference category for software vendor comparisons.

Covariate	Coefficient	Standard Error	95% Lower	95% Upper	*p*-Value
**Intercept**	−17.077	8.885	−34.492	0.339	0.055
**Software: Non-GE**	1.282	0.544	0.214	2.349	0.019
**Age**	−0.224	0.059	−0.341	−0.108	<0.001
**Males**	0.014	0.019	−0.024	0.052	0.465
**BMI**	−0.901	0.291	−1.471	−0.331	0.002
**Alcoholic etiology**	−0.006	0.022	−0.048	0.037	0.794
**MELD score**	0.466	0.076	0.317	0.616	<0.001
**Diabetes**	0.450	0.096	0.262	0.638	<0.001
**HR**	−0.156	0.036	−0.226	−0.085	<0.001
**SBP**	0.379	0.085	0.212	0.546	<0.001
**BB**	0.016	0.023	−0.029	0.061	0.486

**Table 8:** Results of random-effects meta-regression analyses examining potential sources of between-study variability in LV-GCS differences between cirrhotic patients and healthy controls. Regression coefficients represent the estimated change in effect size associated with each moderator variable. Corresponding 95% confidence intervals (95% CIs) and *p* values are reported for all covariates. GE served as the reference category for software vendor analyses.

Covariate	Coefficient	Standard Error	95% Lower	95% Upper	*p*-Value
**Intercept**	7.171	15.210	−22.640	36.982	0.637
**Software: Non-GE**	0.327	1.218	−2.061	2.715	0.788
**Age**	−0.092	0.169	−0.422	0.239	0.587
**Males**	−0.040	0.059	−0.155	0.075	0.492
**MELD score**	0.018	0.213	−0.400	0.436	0.933
**HR**	0.000	0.156	−0.306	0.306	0.999

**Table 9:** Results of random-effects meta-regression analyses examining potential sources of between-study variability in LV-GRS differences between cirrhotic patients and healthy controls.

Covariate	Coefficient	Standard Error	95% Lower	95% Upper	*p*-Value
**Intercept**	−24.825	16.266	−56.704	7.056	0.127
**Software: Non-GE**	0.505	0.571	−0.615	1.625	0.377
**Age**	0.124	0.097	−0.065	0.313	0.197
**BMI**	0.633	0.500	−0.347	1.613	0.205
**MELD score**	0.140	0.133	−0.121	0.400	0.293

## Data Availability

The dataset generated from the extraction of data from the included studies will be made openly accessible through Zenodo (https://zenodo.org; accessed on 7 May 2026).

## Supplementary Materials

The following supporting information can be downloaded at https://www.mdpi.com/article/10.3390/jcm15135139/s1. File S1: PRISMA 2020 checklist; File S2: The full INPLASY record [1,2,3,4,5,6,17,18,19,20,21,22,23,24,25,26,27,28,29,30,31,32,33,34,35,36,38] (https://inplasy.com/inplasy-2026-5-0085/, accessed on 15 May 2026); File S3: Quality appraisal of the studies included in the systematic review [17,18,19,20,21,22,23,24,25,26,27,28,29,30,31,32,33,34,35,36] according to the National Institutes of Health (NIH) Quality Assessment Tool for Observational Cohort and Cross-Sectional Studies [38]. Q1–Q14 correspond to the individual NIH methodological domains evaluating study objectives, population definition, participation rate, exposure and outcome assessment, follow-up, and statistical analyses. Coding was assigned as follows: Y = yes; N = no; NR = not reported; CD = cannot determine; NA = not applicable. Overall methodological quality was expressed as the total number of “Yes” responses, followed by the final qualitative judgement (Good, Fair, or Poor).

## Abbreviations

2D-STE, two-dimensional speckle-tracking echocardiography; ACEi/ARBs, angiotensin-converting enzyme inhibitors/angiotensin receptor blockers; ALT, alanine aminotransferase; AP, alkaline phosphatase; AST, aspartate aminotransferase; BB, beta-blockers; BMI, body mass index; BSA, body surface area; CI, cardiac index; CMR, cardiac magnetic resonance; CMR-FT, cardiac magnetic resonance feature tracking; CRP, C-reactive protein; DBP, diastolic blood pressure; E/A, ratio between early and late transmitral diastolic velocities; E/e′, ratio between early transmitral flow velocity and early diastolic mitral annular velocity; ECV, extracellular volume fraction; GE, General Electric Healthcare; GGT, gamma-glutamyl transferase; GI, gastrointestinal; Hb, hemoglobin; HCC, hepatocellular carcinoma; HR, heart rate; HS-troponin, high-sensitivity troponin; INR, international normalized ratio; IVS, interventricular septum thickness; LA, left atrial; LAScd, left atrial conduit strain; LASct, left atrial contraction strain; LASr, left atrial reservoir strain; LAVi, left atrial volume index; LGE, late gadolinium enhancement; LVEDD, left ventricular end-diastolic diameter; LVEDV, left ventricular end-diastolic volume; LVEF, left ventricular ejection fraction; LVESD, left ventricular end-systolic diameter; LVESV, left ventricular end-systolic volume; LV-GCS, left ventricular global circumferential strain; LV-GLS, left ventricular global longitudinal strain; LV-GRS, left ventricular global radial strain; LVMi, left ventricular mass index; MASLD, metabolic dysfunction-associated steatotic liver disease; MELD, Model for End-Stage Liver Disease; NASH, non-alcoholic steatohepatitis; NS, not specified; NT-proBNP, N-terminal pro-B-type natriuretic peptide; PBC, primary biliary cholangitis; PLTs, platelets; PSC, primary sclerosing cholangitis; PT, prothrombin time; PW, posterior wall thickness; QTc, corrected QT interval; RAAS, renin–angiotensin–aldosterone system; RAScd, right atrial conduit strain; RASct, right atrial contraction strain; RASr, right atrial reservoir strain; RAVi, right atrial volume index; RV, right ventricle; RVEDV, right ventricular end-diastolic volume; RVEF, right ventricular ejection fraction; RVESV, right ventricular end-systolic volume; RV-FWLS, right ventricular free-wall longitudinal strain; RV-GCS, right ventricular global circumferential strain; RV-GLS, right ventricular global longitudinal strain; RV-GRS, right ventricular global radial strain; RV–PA, right ventricular–pulmonary arterial; RWT, relative wall thickness; SBP, systolic blood pressure; sPAP, systolic pulmonary artery pressure; STE, speckle-tracking echocardiography; SV, stroke volume; TAPSE, tricuspid annular plane systolic excursion; TIPS, transjugular intrahepatic portosystemic shunt.
