# Supplementary material for: Biventricular Systolic Function and Myocardial Deformation in Liver Cirrhosis: A Systematic Review and Meta-Analysis of Speckle-Tracking Echocardiography and Cardiac Magnetic Resonance Feature Tracking Studies
Source: J Clin Med. 2026 Jul 1;15(13):5139. doi: 10.3390/jcm15135139 (PMC13363545; doi:10.3390/jcm15135139)
Supplement: Supplementary file 1 [file jcm-15-05139-s001.zip › File S2.pdf]

Sonaglioni et al. INPLASY protocol 202650085. doi:10.37766/inplasy2026.5.0085 Downloaded from https://inplasy.com/inplasy-2026-5-0085/

INPLASY

INPLASY202650085

doi: 10.37766/inplasy2026.5.0085

Received: 15 May 2026

Published: 15 May 2026

Corresponding author:
Andrea Sonaglioni

sonaglioniandrea@gmail.com

Author Affiliation:
IRCCS MultiMedica.

Biventricular Systolic Function and Myocardial Deformation in Liver Cirrhosis: A Systematic Review and Meta-Analysis of Speckle-Tracking Echocardiography and Cardiac Magnetic Resonance Feature Tracking Studies

Sonaglioni, A; Gramaglia, GF; Canova, L; Cerini, F; Rumi, MG; Nicolosi, GL; Baravelli, M; Lombardo, M.

ADMINISTRATIVE INFORMATION

Support - Ministero della Salute Ricerca Corrente.

Review Stage at time of this submission - Completed but not published.

Conflicts of interest - None declared.

INPLASY registration number: INPLASY202650085

Amendments - This protocol was registered with the International Platform of Registered Systematic Review and Meta-Analysis Protocols (INPLASY) on 15 May 2026 and was last updated on 15 May 2026.

INTRODUCTION

Review question / Objective The aim of the present systematic review and meta-analysis was to comprehensively evaluate the impact of liver cirrhosis on biventricular systolic function assessed by advanced deformation imaging techniques. Specifically, we analyzed differences in left ventricular ejection fraction (LVEF), left ventricular global longitudinal strain (LV-GLS), left ventricular global circumferential strain (LV-GCS), left ventricular global radial strain (LV-GRS), right ventricular global longitudinal strain (RV-GLS), and right ventricular ejection fraction (RVEF) between cirrhotic patients and control subjects using data derived from two-dimensional speckle-tracking echocardiography (2D-STE) and/or cardiac magnetic resonance feature tracking (CMR-FT) studies. In addition, we sought to provide pooled descriptive estimates of clinical, laboratory, echocardiographic, and CMR characteristics of the analyzed cirrhotic populations in order to better contextualize myocardial mechanical alterations across different stages and etiologies of liver disease.

Rationale Several studies have evaluated myocardial deformation in patients with liver cirrhosis using 2D-STE and/or CMR-FT [17-36], reporting heterogeneous findings regarding both left and right ventricular systolic performance. In particular, LV-GLS has been increasingly investigated as a potential marker of subclinical systolic dysfunction in cirrhotic patients, although available studies have reported inconsistent results, ranging from impaired longitudinal deformation to apparently supranormal strain values likely related to hyperdynamic circulation and altered loading conditions. Similarly, limited evidence exists regarding LV-GCS, LV-GRS, RV-GLS, and RVEF, especially when assessed using CMR-based techniques. Furthermore, substantial heterogeneity among studies exists in terms of imaging modality, software vendor, cirrhosis etiology, disease severity, sample size, and inclusion of control populations.

A comprehensive synthesis integrating conventional systolic indices and multidirectional myocardial deformation parameters in cirrhotic patients is currently lacking. In addition, the relative contribution of echocardiographic and CMR-

derived strain analysis to the characterization of cirrhotic myocardial involvement remains incompletely defined.

References
17. Altekin, R.E.; Caglar, B.; Karakas, M.S.; Ozel, D.; Deger, N.; Demir, I. Evaluation of subclinical left ventricular systolic dysfunction using two-dimensional speckle-tracking echocardiography in patients with non-alcoholic cirrhosis. Hellenic J. Cardiol. 2014, 55, 402–410.
18. https://doi.org/10.1186/s12968-015-0157-6.
19. https://doi.org/10.1016/j.jjcc.2015.08.001.
20. https://doi.org/10.1002/hep.29104.
21. https://doi.org/10.1080/19932820.2017.1283162.
22. https://doi.org/10.1111/apt.14934.
23. https://doi.org/10.3390/jcm8091285.
24. https://doi.org/10.15171/jcvtr.2019.22.
25. https://doi.org/10.5543/tkda.2019.94728.
26. https://doi.org/10.1186/s12968-020-00622-2.
27. https://doi.org/10.1148/radiol.2020201057.
28. https://doi.org/10.3390/jcm10050897.
29. https://doi.org/10.1007/s10554-021-02260-w.
30. https://doi.org/10.3389/fgstr.2022.860800.
31. https://doi.org/10.1007/s12664-022-01277-w.
32. https://doi.org/10.1016/j.amjcard.2023.09.069.
33. https://doi.org/10.1111/liv.15714.
34. https://doi.org/10.1007/s00330-025-11710-1.
35. https://doi.org/10.1177/03000605251411145.
36. https://doi.org/10.3390/jcm15072791.

Condition being studied Liver cirrhosis is a complex multisystem disease characterized not only by progressive hepatic dysfunction and portal hypertension, but also by significant cardiovascular alterations that may substantially influence clinical status, prognosis, and perioperative risk, particularly in candidates for liver transplantation [1-3]. Among these extrahepatic manifestations, cirrhotic cardiomyopathy (CCM) has emerged as a clinically relevant entity characterized by impaired cardiac reserve, subclinical systolic and diastolic dysfunction, electrophysiological abnormalities, and altered myocardial responsiveness to physiological or pharmacological stress despite apparently preserved resting cardiac performance [4-6].

References
1. Carvalho, M.V.H.; Kroll, P.C.; Kroll, R.T.M.; Carvalho, V.N. Cirrhotic cardiomyopathy: the liver affects the heart. Braz. J. Med. Biol. Res. 2019, 52, e7809. https://doi.org/10.1590/1414-431X20187809.
2. Gananandan, K.; Wiese, S.; Møller, S.; Mookerjee, R.P. Cardiac dysfunction in patients with cirrhosis and acute decom-pensation. Liver Int. 2024, 44, 1832–1841. https://doi.org/10.1111/liv.15896.
3. Ali, S.A.; Frick, K. The Cardiohepatic Axis in Cirrhosis. JACC Basic Transl. Sci. 2025, 10, 101314. https://doi.org/10.1016/j.jacbts.2025.101314.
4. Liu, H.; Naser, J.A.; Lin, G.; Lee, S.S. Cardiomyopathy in cirrhosis: From pathophysiology to clinical care. JHEP Rep. 2023, 6, 100911. https://doi.org/10.1016/j.jhepr.2023.100911.
5. Lupu, D.; Scârneciu, C.C.; Ţînţ, D.; Tudoran, C. Cirrhotic Cardiomyopathy: Bridging Hepatic and Cardiac Pathophysio-logy in the Modern Era. J. Clin. Med. 2025, 14, 5993. https://doi.org/10.3390/jcm14175993.
6. Gill, M.; Koshy, A.N.; Ramachandran, J.; McCaughan, G.W.; Majumdar, A. Cirrhotic cardiomyopathy: pathophysiology, assessment, and implications for liver transplantation. Ther. Adv. Gastroenterol. 2026, 19, 17562848261423358. https://doi.org/10.1177/17562848261423358.

METHODS

Search strategy A comprehensive literature search was independently conducted by two investigators to identify all studies evaluating myocardial systolic function and deformation parameters in patients with liver cirrhosis using 2D-STE and/or CMR-FT. Electronic databases including PubMed, Scopus, and EMBASE were systematically searched from database inception to May 2026.
The search strategy combined Medical Subject Headings (MeSH) terms and free-text keywords related to liver cirrhosis, end-stage liver disease, myocardial deformation imaging, and ventricular systolic function. Search terms included combinations of “liver cirrhosis”, “cirrhotic cardiomyopathy”, “end-stage liver disease”, “global longitudinal strain”, “GLS”, “myocardial strain”, “left ventricular strain”, “right ventricular strain”, “speckle tracking echocardiography”, “2D-STE”, “cardiac magnetic resonance”, “CMR”, “feature tracking”, “CMR-FT”, “left ventricular ejection fraction”, and “right ventricular ejection fraction”. No restrictions regarding language, year of publication, or geographic region were applied.
Because the primary aim of the study was to comprehensively assess both conventional and deformation-derived indices of biventricular systolic function, data regarding LVEF, LV-GLS, LV-GCS, LV-GRS, RV-GLS, and RVEF were extracted from all eligible studies whenever available. No separate search strategy was performed for individual strain parameters or ventricular function indices, as all variables were analyzed within the same cirrhotic populations.
In addition, the reference lists of all included articles and relevant review papers were manually screened to identify potentially eligible studies not captured through the electronic database search. Any disagreement between investigators during study screening and selection was resolved by discussion and consensus, with involvement of a third reviewer when necessary.

Sonaglioni et al. INPLASY protocol 202650085. doi:10.37766/inplasy2026.5.0085 Downloaded from https://inplasy.com/inplasy-2026-5-0085/

Participant or population Patients with liver cirrhosis who underwent biventricular assessment by 2D-STE and/or CMR-FT.

Intervention Biventricular assessment by 2D-STE and/or CMR-FT.

Comparator Healthy controls.

Study designs to be included Observational Cohort and Cross-Sectional Studies.

Eligibility criteria Studies were considered eligible if they had an observational design, including prospective or retrospective cohort studies and cross-sectional investigations, and evaluated left and/or right ventricular systolic function in adult patients with liver cirrhosis or end-stage liver disease using 2D-STE and/or CMR-FT. Eligible studies were required to provide extractable quantitative data regarding at least one of the following parameters: LVEF, LV-GLS, LV-GCS, LV-GRS, RV-GLS, or RVEF. Continuous variables had to be reported as mean ± standard deviation, median with dispersion measures, or in a format suitable for statistical reconstruction.
Studies including a healthy control group were considered eligible for quantitative meta-analysis. Investigations without a control population were retained for the systematic review and descriptive pooled analyses but were excluded from comparative meta-analytic synthesis.
Both echocardiographic and CMR-derived deformation analyses were considered eligible because the aim of the study was to comprehensively assess myocardial mechanics across different imaging modalities. Accordingly, studies using 2D-STE, stress-STE, or CMR-FT were included. In contrast, studies using non-standardized or experimental deformation techniques, including strain-encoded imaging (SENC), myocardial tagging without feature-tracking analysis, or other investigational approaches lacking comparability with STE or CMR-FT methodology, were excluded.
Studies performed in pediatric populations, animal models, or experimental preclinical settings were excluded. Similarly, studies enrolling mixed populations without separately extractable cirrhotic data were not considered eligible. To minimize confounding effects on myocardial function, studies focused primarily on patients with overt structural heart disease, significant valvular disease, known cardiomyopathies, congenital heart disease, or severe pulmonary hypertension unrelated to liver disease were also excluded whenever these populations could not be clearly separated from the cirrhotic cohort.
Non-original articles including editorials, narrative reviews, conference abstracts, case reports, expert opinions, and guidelines were excluded. In addition, studies lacking sufficient quantitative imaging data for pooled analysis were not included in the meta-analysis.

Information sources Electronic databases including PubMed, Scopus, and EMBASE were systematically searched from database inception to May 2026.

Main outcome(s) The primary quantitative analyses aimed to evaluate the effect of liver cirrhosis on conventional and deformation-derived indices of biventricular systolic function. Separate meta-analyses were therefore performed for LVEF, LV-GLS, LV-GCS, LV-GRS, RV-GLS, and RVEF. Comparative pooled analyses between cirrhotic patients and controls were conducted using standardized mean differences (SMDs) with corresponding 95% confidence intervals (CIs). SMDs were selected to account for differences in study dispersion and variability across imaging modalities, software vendors, and acquisition protocols.

Additional outcome(s) To compare 2D-STE vs. CMR-FT studies.

Data management Two investigators independently screened all retrieved records by title and abstract, followed by full-text evaluation of potentially eligible articles according to the predefined inclusion and exclusion criteria. Any disagreement regarding study eligibility was resolved through discussion and consensus, with consultation of a third reviewer when necessary.
Data extraction was independently performed by two investigators using a standardized data collection form specifically developed for the present review. Extracted study-level variables included first author, publication year, country, study design, imaging modality, software vendor, and study population size.
Demographic and clinical variables were systematically collected when available, including age, sex distribution, body mass index (BMI), body surface area (BSA), cardiovascular risk factors, blood pressure values, heart rate, smoking status, cirrhosis etiology, Child–Pugh score, MELD score, prevalence of ascites, encephalopathy, hepatopulmonary syndrome, hepatorenal syndrome, gastrointestinal bleeding, and ongoing medical therapies. Laboratory parameters including hemoglobin, platelet count, creatinine, sodium, bilirubin, albumin, liver enzymes, NT-proBNP, high-sensitivity troponin, C-reactive

Sonaglioni et al. INPLASY protocol 202650085. doi:10.37766/inplasy2026.5.0085 Downloaded from https://inplasy.com/inplasy-2026-5-0085/

protein, and coagulation indices were additionally extracted whenever reported.

Conventional echocardiographic parameters were collected to characterize cardiac geometry, systolic performance, and diastolic function in cirrhotic patients and control subjects. Extracted variables included interventricular septal thickness, posterior wall thickness, left ventricular end-diastolic and end-systolic diameters and volumes, relative wall thickness, left ventricular mass index, stroke volume, cardiac index, left atrial diameter and volume index, right ventricular dimensions, tricuspid annular plane systolic excursion (TAPSE), systolic pulmonary artery pressure (sPAP), TAPSE/sPAP ratio, transmitral E/A ratio, and E/e' ratio.

Strain-derived myocardial deformation parameters obtained by 2D-STE and/or CMR-FT were systematically extracted. These included LV-GLS, LV-GCS, LV-GRS, RV-GLS, RV-free wall longitudinal strain (FWLS), left atrial strain indices, and right atrial strain parameters when available. Right and left ventricular ejection fraction values derived from echocardiography and/or CMR were additionally collected.

CMR-derived structural and tissue characterization variables were also recorded whenever available, including left and right ventricular volumes, myocardial mass, late gadolinium enhancement (LGE), native T1 and T2 relaxation times, and extracellular volume fraction (ECV).

For consistency of presentation and easier interpretation, strain parameters originally reported as negative percentages were uniformly expressed throughout tables and meta-analytic analyses as absolute positive values, without modifying relative intergroup differences or statistical significance. When quantitative values were available only in graphical format, numerical data were extracted using dedicated digital plot analysis software. All extracted variables were independently cross-checked by both reviewers, and any discrepancy was resolved through re-evaluation of the original manuscript until consensus was reached.

Quality assessment / Risk of bias analysis The methodological quality of the included studies and the potential risk of bias were independently evaluated by two investigators using the National Institutes of Health (NIH) Quality Assessment Tool for Observational Cohort and Cross-Sectional Studies [38]. This instrument assesses several methodological aspects including study population definition, participant selection, exposure and outcome assessment, reproducibility of measurements, handling of confounding variables, statistical methodology, and adequacy of data reporting.

Each study was evaluated across 14 predefined domains according to NIH recommendations, with each item categorized as "Yes", "No", "Cannot Determine", "Not Reported", or "Not Applicable". Positive responses were considered indicative of satisfactory methodological quality, whereas items judged as not applicable were excluded from the overall scoring process. A cumulative quality score was calculated on the basis of the number of fulfilled criteria.

Overall study quality was categorized using predefined thresholds. Studies satisfying 9–14 items were considered of good methodological quality, those fulfilling 5–8 criteria were classified as fair quality, whereas studies meeting fewer than 5 criteria were considered poor quality. Final quality categorization was based not only on the numerical score, but also on reviewer judgment regarding the clinical relevance of specific methodological limitations.

Any discrepancy between reviewers regarding individual domains or final quality classification was resolved through joint reassessment of the original articles until consensus was achieved. Risk-of-bias findings were subsequently summarized using graphical representations and tabulated quality assessment summaries.

Rererences:
38. Ma, L.L.; Wang, Y.Y.; Yang, Z.H.; Huang, D.; Weng, H.; Zeng, X.T. Methodological quality (risk of bias) assessment tools for primary and secondary medical studies: what are they and which is better? Mil. Med. Res. 2020, 7, 7. https://doi.org/10.1186/s40779-020-00238-8.

Strategy of data synthesis To provide an overall characterization of cirrhotic populations and control subjects across the included studies, pooled descriptive estimates were calculated using study-level data. Continuous variables were summarized as weighted medians with weighted interquartile ranges (Q1–Q3), using study sample size as weighting factor. Since most studies reported variables as mean ± standard deviation, study-level distributions were approximated assuming normal distribution in order to derive pooled weighted medians and dispersion measures. These pooled estimates were intended to provide a descriptive overview of the analyzed populations rather than formal patient-level inferential statistics. Accordingly, reported p values for pooled descriptive comparisons should be considered exploratory and interpreted cautiously.

The primary quantitative analyses aimed to evaluate the effect of liver cirrhosis on conventional and deformation-derived indices of biventricular systolic function. Separate meta-analyses were therefore performed for LVEF, LV-GLS, LV-GCS, LV-

Sonaglioni et al. INPLASY protocol 202650085. doi:10.37766/inplasy2026.5.0085 Downloaded from https://inplasy.com/inplasy-2026-5-0085/

GRS, RV-GLS, and RVEF. Comparative pooled analyses between cirrhotic patients and controls were conducted using standardized mean differences (SMDs) with corresponding 95% confidence intervals (CIs). SMDs were selected to account for differences in study dispersion and variability across imaging modalities, software vendors, and acquisition protocols.

When studies reported continuous variables as medians with dispersion measures, mean values and standard deviations were reconstructed using validated statistical conversion methods before inclusion in meta-analytic pooling. Similarly, when quantitative data were available only graphically, numerical values were extracted using dedicated digital plot analysis software.

Fixed-effects or random-effects models were selected according to the degree of between-study heterogeneity. Random-effects models based on the DerSimonian–Laird approach were preferentially adopted in the presence of relevant clinical or methodological heterogeneity, whereas fixed-effects models were used when heterogeneity was negligible. Statistical heterogeneity was assessed using Cochran's Q statistic and quantified with the I^2 index, with I^2 values of approximately 25%, 50%, and 75% considered indicative of low, moderate, and high heterogeneity, respectively.

Sensitivity analyses were systematically performed for each pooled outcome using a leave-one-out approach to evaluate the robustness and stability of pooled estimates for LVEF, LV-GLS, LV-GCS, LV-GRS, RV-GLS, and RVEF. Publication bias and small-study effects were explored through visual inspection of funnel plots and formally assessed using Egger's regression asymmetry test whenever appropriate.

Meta-regression analyses were additionally performed in the presence of random-effects models to investigate potential sources of heterogeneity among studies. Predefined study-level covariates included age, sex distribution, body mass index, MELD score, Child–Pugh score, heart rate, blood pressure values, imaging modality, and software vendor whenever sufficient data were available. Regression coefficients, standard errors, 95% confidence intervals, and two-sided p values were calculated for each moderator variable.

All statistical analyses were performed using Comprehensive Meta-Analysis software (version 3.0; Biostat, Englewood, NJ, USA). All statistical tests were two-sided, and a p value < 0.05 was considered statistically significant.

Subgroup analysis 2D-STE vs. CMR-FT studies.

Sensitivity analysis Sensitivity analyses were systematically performed for each pooled outcome using a leave-one-out approach to evaluate the robustness and stability of pooled estimates for LVEF, LV-GLS, LV-GCS, LV-GRS, RV-GLS, and RVEF. Publication bias and small-study effects were explored through visual inspection of funnel plots and formally assessed using Egger's regression asymmetry test whenever appropriate.

Language restriction No.

Country(ies) involved Italy.

Keywords liver cirrhosis; cirrhotic cardiomyopathy; speckle-tracking echocardiography; cardiac magnetic resonance; feature tracking; myocardial strain; global longitudinal strain.

Contributions of each author

Author 1 - Andrea Sonaglioni - Author 1 drafted the manuscript.
Email: sonaglioniandrea@gmail.com
Author 2 - Giulio Francesco Gramaglia - The author contributed to the Search Strategy.
Email: giulio.gramaglia@unimi.it
Author 3 - Lorenzo Canova - The author read, provided feedback and approved the final manuscript.
Email: lorenzo.canova@multimedica.it
Author 4 - Federica Cerini - The author read, provided feedback and approved the final manuscript.
Email: federica.cerini@multimedica.it
Author 5 - Maria Grazia Rumi - The author read, provided feedback and approved the final manuscript.
Email: mariagrazia.rumi@unmimi.it
Author 6 - Gian Luigi Nicolosi - The author read, provided feedback and approved the final manuscript.
Email: gianluigi.nicolosi@gmail.com
Author 7 - Massimo Baravelli - The author read, provided feedback and approved the final manuscript.
Email: massimo.baravelli@multimedica.it
Author 8 - Michele Lombardo - The author read, provided feedback and approved the final manuscript.
Email: michele.lombardo@multimedica.it

Sonaglioni et al. INPLASY protocol 202650085. doi:10.37766/inplasy2026.5.0085 Downloaded from https://inplasy.com/inplasy-2026-5-0085/
