# Supplementary material for: Biventricular Systolic Function and Myocardial Deformation in Liver Cirrhosis: A Systematic Review and Meta-Analysis of Speckle-Tracking Echocardiography and Cardiac Magnetic Resonance Feature Tracking Studies
Source: J Clin Med. 2026 Jul 1;15(13):5139. doi: 10.3390/jcm15135139 (PMC13363545; doi:10.3390/jcm15135139)
Supplement: Supplementary file 1 [file jcm-15-05139-s001.zip › File S3.pdf]

Study	Q1	Q2	Q3	Q4	Q5	Q 6	Q7	Q 8	Q9	Q1 0	Q1 1	Q1 2	Q13	Q1 4	Overall
Altekin R.E. [17]	Y	Y	NR	Y	NR	Y	Y	Y	Y	N	Y	NR	NA	Y	9 (Good)
Sampaio F. 18]	Y	Y	NR	Y	NR	Y	Y	Y	Y	Y	Y	Y	NA	Y	11 (Good)
Chen Y. ([19]	Y	Y	NR	Y	NR	Y	Y	Y	Y	Y	Y	NR	Y	Y	11 (Good)
Yotti R. [20]	Y	Y	NR	Y	NR	Y	Y	Y	Y	Y	Y	NR	NA	Y	10 (Good)
Hammami R. [21]	Y	Y	NR	Y	NR	Y	Y	Y	Y	N	Y	Y	NA	N	9 (Fair)
Novo G. [22]	Y	Y	NR	Y	NR	Y	Y	Y	Y	Y	Y	NR	Y	Y	11 (Good)
Zhang K. [23]	Y	Y	NR	Y	NR	Y	Y	Y	Y	N	Y	Y	NA	Y	10 (Good)
Zamirian M. [24]	Y	CD	NR	CD	NR	Y	Y	N	CD	N	Y	NR	NA	N	4 (Poor)
Demirtaş Inci S. [25]	Y	Y	NR	Y	NR	Y	Y	Y	Y	N	Y	NR	NA	N	8 (Fair)
Kim H.M. [26]	Y	Y	NR	Y	NR	Y	Y	Y	Y	Y	Y	NR	Y	Y	11 (Good)
Isaak A. [27]	Y	Y	NR	Y	NR	Y	Y	Y	Y	N	Y	NR	NA	Y	9 (Good)
von Köckritz F. [28]	Y	Y	NR	Y	NR	Y	Y	Y	Y	N	Y	NR	CD	Y	9 (Fair)
Soulaidopoulos S. [29]	Y	Y	Y	Y	NR	Y	Y	Y	Y	N	Y	NR	NA	Y	10 (Good)
Jansen C. [30]	Y	Y	N	CD	NR	Y	Y	Y	Y	Y	Y	NR	Y	Y	10 (Fair)
Poojary M.S. [31]	Y	Y	NR	Y	NR	Y	Y	Y	Y	N	Y	Y	NA	N	9 (Fair)
Luo Y. [32]	Y	Y	NR	Y	NR	Y	Y	Y	Y	N	Y	NR	Y	Y	10 (Good)
Skouloudi M. [33]	Y	Y	NR	Y	NR	Y	Y	Y	Y	N	Y	NR	CD	Y	9 (Good)
Erley J. [34]	Y	Y	NR	Y	NR	Y	Y	Y	Y	N	Y	NR	NA	Y	9 (Good)
Yang X. [35]	Y	Y	NR	Y	NR	Y	Y	Y	Y	N	Y	NR	NA	N	8 (Fair)
Radu T. [36]	Y	Y	NR	Y	NR	Y	Y	Y	Y	N	Y	NR	Y	Y	10 (Fair)
